# Shallow water sea slugs (Gastropoda: Heterobranchia) from the northwestern coast of the Sea of Japan, north of Peter the Great Bay, Russia

**DOI:** 10.7717/peerj.2774

**Published:** 2016-12-08

**Authors:** Anton Chichvarkhin

**Affiliations:** National Scientific Center of Marine Biology, Far Eastern Branch of Russian Academy of Sciences, Vladivostok, Russia; Far Eastern Federal University, Vladivostok, Russia

**Keywords:** Opisthobranchia, Sacoglossa, Thecosomata, Fauna, Nudibranchia, *Cadlina olgae*, Cephalaspidea, Sea of Japan, Biogeography

## Abstract

The coast of northern Primorye region, north of Peter the Great Bay has been sparsely studied in regards to its molluscan fauna, with just a few works reviewing the distribution of local mollusks. This work presents a survey of the shallow water heterobranch sea slugs currently occurring around Kievka Bay to Oprichnik Bay, Russia. Thirty-nine species of sea slugs were found in this study and the new species *Cadlina olgae* sp. nov., described herein. Most (24) of the species occurring in the area have widespread ranges in the northern Pacific Ocean. The eight species are endemic for the Sea of Japan and adjacent part of the Sea of Okhotsk. Seven other occur also in northern Atlantic and Arctic waters. Thirteen found species are not known from Peter the Great Bay but known from adjacent northern Pacific waters. The finding of a previously undescribed species emphasizes the need of further surveys, particularly in subtidal and deeper waters, in order to improve the knowledge on this neglected fauna in Primorye.

## Introduction

The Heterobranch sea slugs of Russian Far East have been sparsely studied; the best-studied area is Peter the Great Bay, the southernmost Russian shore in Asia, although the fauna of this bay has not been studied untill first half of the 20th century. The studies in this area revealed a number of species, many of them were new for the Russian fauna, and a number of new taxa were described (e.g., Volodchenko, 1941; Minichev, 1976; Minichev, 1971; Minichev, Roginskaya & Slavoshevskaya, 1971; Slavoshevskaya, 1971; Martynov, 1992; Martynov, 1998a; Martynov, 2002; Martynov, 2003; Chernyshev, 2008; Chernyshev, 2014; Chaban & Chernyshev, 2009; Chaban & Chernyshev, 2014; Chernyshev & Chaban, 2010; Martynov, Sanamyan & Korshunova, 2015). However, the coastline located north off Peter the Great Bay remains almost totally unattended by malacologists besides a few new species descriptions (Volodchenko, 1941; Martynov, 2002). More recently, we have reported several new species for Sea of Japan and the Russian fauna from Rudnaya and Vladimir Bays (Chichvarkhin, Chichvarkhina & Chernyshev, 2015; Chichvarkhin, Chichvarkhina & Kartavtsev, 2016a; Chichvarkhin et al., 2016b; Breslau, Valdés & Chichvarkhin, in press; Ekimova et al., 2016).

The present study provides records of sea slugs found in shallow waters (above 30 m depth) between Kievka Bay (42.85°N) and Oprichnik Bay (44,45°N), Primorskiy Krai, Russia. The coast of this area consists of rocky formations with sparse sandy beaches and a quite narrow intertidal zone. Rocky platforms and boulder fields are common; however, some sheltered areas have open sandy beaches, usually exposed to strong surf (e.g., Rudnaya, Kievka Bays). The goal of this preliminary study is to contribute to the knowledge of the molluscan fauna in Russian Far East, particularly providing a tool useful for identification of live animals in the field.

## Materials and Methods

**Figure 1:**
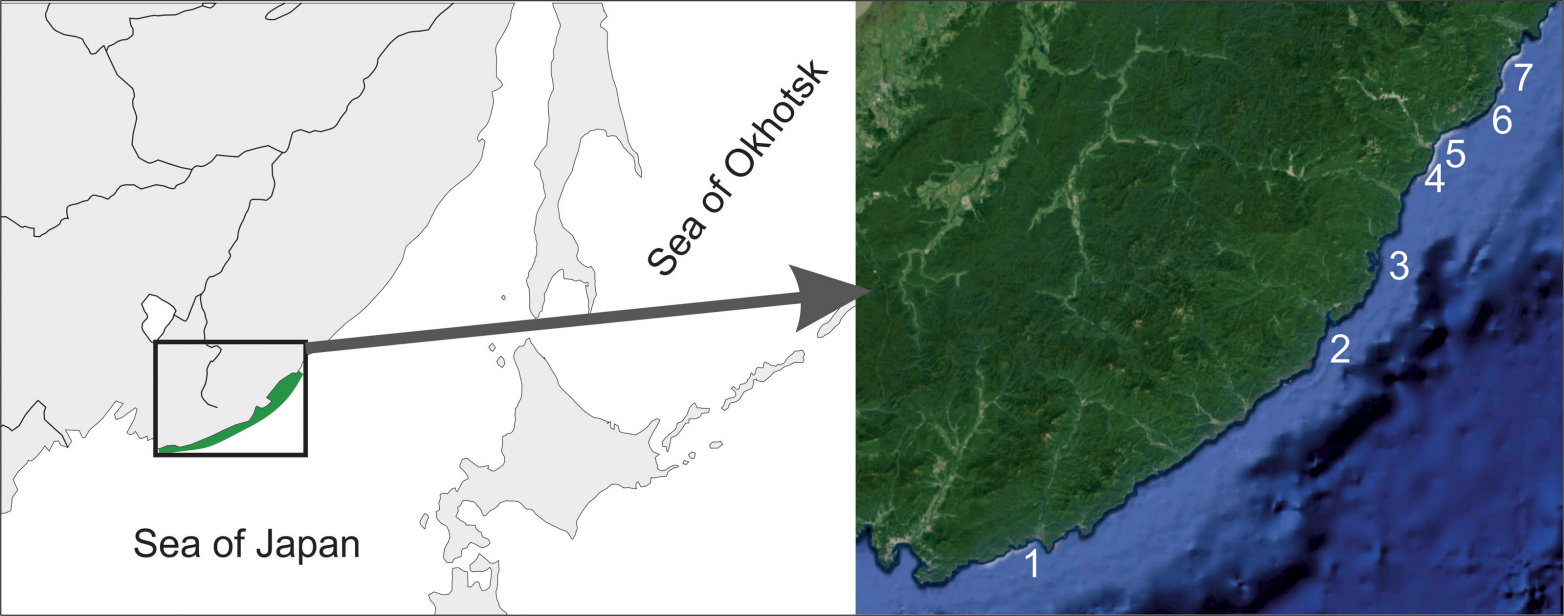
Surveyed area map. 1–Kievka Bay (42.84°N 133.65°E), 2–Olga Bay (43.74°N 135.27°E), 3–Vladimir Bay (43.91°N 135.50°E), 4–Dva Brata, Senkina Shapka (44.33°N 135.84°E), 5–Rudnaya Bay, Brynner Cape (44.36°N 135.80°E), 6–Tretya Langou, Kamenka Bay (44.42°N 135.94°E), 7–Oprichnik Bay (44.45°N 136.00°E).

The material examined was collected during the summers of 2012–2016 in several locations between Kievka and Oprichnik Bays (Fig. 1) of the northwestern Sea of Japan, Primorskiy Krai, Russia. All the collecting was made manually by SCUBA diving, mostly on rocky walls, platforms, and the pinnacles. Four specimens of *Cadlina laevis* collected in the White Sea Biological Station, Moscow University, White Sea, Russia were also examined. The specimens were deposited in the collections of the Museum of A.V. Zhirmunsky Institute of Marine Biology, Russian Academy of Sciences (MIMB) and Zoological Museum, Moscow State University (ZMMU).

Field study permits were not required for this study and none of the species studied herein are currently under legal protection. All the collected specimens were preserved in 95% ethanol. Photography was performed with a Nikon D300 or D810 cameras with a Nikkor 105/2.8G lens in appropriate Sea&Sea housings with Sea&Sea YS-D1 strobes when used underwater. All sizes given are living measurements, radular features were examined after carbon coating by field emission scanning electron microscope Zeiss Sigma using a ETSE detector at EHT 10 kV. Color plates were composed with Adobe Photoshop software and original colors of the images were not modified.

In order to characterize genetically and barcode the new species of *Cadlina*, DNA extraction was performed using DNEasy kit (Qiagen). Folmer’s universal COI (Folmer et al., 1994), and 16S rRNA gene fragment primers (Palumbi, 1996) were used to amplify the region of interest for three specimens of *Cadlina olgae* sp.n. and two specimens in *C. laevis*. For two specimens of *Limacina helicina*, the COI fragment was amplified only. The master mix (for each sample) was prepared using 34.75 mL H_2_O, 5.00 mL PCR Buffer (Evrogen, Moscow, Russia), 5.00 mL 25 mM MgCl_2_, 1.00 mL 40 mM dNTPs, 1.00 mL 10 mM primer 1, 1.00 mL primer 2, 0.25 mL 5 mg/mL Taq, and 1.00 mL extracted DNA. Reaction conditions were an initial denaturation for 3 min at 95 C, 39 cycles of 1) denaturation for 45 sec at 94 °C, 2) annealing for 45 sec at 50 °C, and 3) elongation for 2 min at 72 °C, and a final elongation for 10 min at 72 °C. PCR products yielding bands of appropriate size (approximately 695 bp in COI, and 421 in 16S) were purified using the Montage PCR Cleanup Kit (Millipore). Cleaned PCR samples were quantified using a NanoDrop 3000 Spectrophotometer (Thermo Scientific). Sequencing was conducted by Sanger ddNTP termination method using BrightDye chemistry (Nimagen) and ABI 3500 Genetic Analyser (Applied Biosystems). The sequences were assembled and edited using BioEdit (Hall, 1999). BioEdit was also used to extract the consensus sequences The sequences used in this study are listed in the Table 1, most of acquired from GenBank sequences were obtained by Johnson (2010).

**Table 1:** Nucleotide sequences used in this study. Marked NCBI numbers indicate data obtained in this study.

Species	Location	Voucher#	COI NCBI#	16S NCBI#
*Cadlina laevis*	Mausunduer, Froya, Norway	CASIZ 182928	EU982716	EU982766
*C. laevis*	Kinkell Braes, Scotland	–	AY345034	–
*C. laevis*	Marstrand, Bohuslan, Sweden	–	AJ223258	AJ225182
*C. laevis*	White Sea	AC23-1	**KX938359**	–
*C. laevis*	White Sea	AC23-2	**KX938360**	–
*C. sp.* 1	Bering Sea	AC17-29	**KX938362**	**KX938358**
*C. sp.* 1	Bering Sea	AC17-28	**KX938361**	**KX938357**
*C. olgae*	Rudnaya Bay	AC16-30	**KX610756**	**KX938355**
*C. olgae*	Rudnaya Bay	AC7-14	**KX610757**	**KX938354**
*C. olgae*	Rudnaya Bay	AC16-31	**KX610758**	**KX938356**
*C. pellucida*	Ilha de Pesequeiro, Portugal	CASIZ 175448	EU982724	EU982774
*C. luteomarginata*	Canada: British Columbia, Bamfield	–	EU982720	EU982770
*C. luteomarginata*	Canada: British Columbia, Bamfield	10BCMOL-00278	KF644272	–
*C. luteomarginata*	Canada: British Columbia, Bamfield	10BCMOL-00358	KF644258	–
*C. luteomarginata*	Bamfield,, British Columbia, Canada	CASIZ 182929	EU982719	EU982769
*C*.aff. *luteomarginata*	Mendocino County, CA, USA	–	EU982721	EU982771
*C*.aff. *luteomarginata*	Canada: Parksville, Vancouver Island, British Columbia	CASIZ 188599A	KM219678	KJ653679
*C. luarna*	Punta Sabana, Costa Rica	CASIZ 175437	EU982718	EU982768
*C. luarna*	Costa Rica	–	EU982717	EU982767
*C. rumia*	Entrade al Parque, Bocas del Toro, Panama	CASIZ 175456	EU982725	EU982775
*C. modesta*	Cayucos, California, USA	CASIZ 182930	EU982723	EU982773
*C. modesta*	Pillar Point, San Mateo County, California, USA	–	EU982722	EU982772
*C. sparsa*	La Jolla, San Diego County, California, USA	CASIZ 182932	EU982726	EU982776
*C. flavomaculata*	Palos Verdes, California, USA	AM C203860	EU982715	EF534041
*C. flavomaculata*	Point Loma, San Diego California, USA	CASIZ 182923	EF535109	EU982764
*C. japonica*	South Korea	CASIZ 182925	–	EU982765
*C. sp.* 2	Cape Peninsula, Cape Province, South Africa	CASIZ 175547	EU982727	EU982777
*Limacina helicina*	Rudnaya Bay	AC6-1	**KX871888**	–
*L. helicina*	Rudnaya Bay	AC6-3	**KX871889**	–
*L. helicina*	Antarctic Ocean	–	KC774084	–
*L. helicina*	Carribean Sea, Yukatan, Belize	–	KC774083	–
*L. helicina*	Arctic Ocean	–	AB859536	–
*L. helicina*	Arctic Ocean: north of Europe	Ga56.2.1	FJ876924	–
*L. helicina*	Pacific Ocean: Prince Williams Sound	Ga56.1.1	FJ876923	–
*L. helicina*	Arctic ocean	–	AB859537	–

The electronic version of this article in Portable Document Format (PDF) will represent a published work according to the International Commission on Zoological Nomenclature (ICZN), and hence the new names contained in the electronic version are effectively published under that Code from the electronic edition alone. This published work and the nomenclatural acts it contains have been registered in ZooBank, the online registration system for the ICZN. The ZooBank LSIDs (Life Science Identifiers) can be resolved and the associated information viewed through any standard web browser by appending the LSID to the prefix http://zoobank.org/. The LSID for this publication is: urn:lsid:zoobank.org:pub:02814E3B-C41F-4AA7-80B9-D4DD2ED73FF2. The online version of this work is archived and available from the following digital repositories: PeerJ, PubMed Central and CLOCKSS.

ABGD method (Puillandre et al., 2012) is based on pairwise distances, detecting the breaks in the distribution referred to as the “barcode gap” (Hebert et al., 2003) without any prior species hypothesis. It is commonly used for species delimitation analyses, including the latest works on molluscan taxa (Jörger et al., 2012; Barco et al., 2013; Krug et al., 2013; Ekimova et al., 2015; Katugin et al., 2015). The ABGD program is available at the web-site http://wwwabi.snv.jussieu.fr/public/abgd/abgdweb.html. We analyzed COI and 16S alignments using uncorrected *p*-distance. The other settings remained as default except the relative gap width (X) was set to 0.9 for 16S dataset.

## Results

**Table:** 

**Systematics**
**Heterobranchia**
**Order Cephalaspidea P. Fischer, 1883**
**Superfamily Philinoidea Gray, 1850 (1815)**
**Family Aglajidae Pilsbry, 1895 (1847)**
**Genus** ***Melanochlamys*** **Cheeseman, 1881**
**Type species** *Melanochlamys cylindrica* Cheeseman, 1881, by original designation.

**Figure 2:**
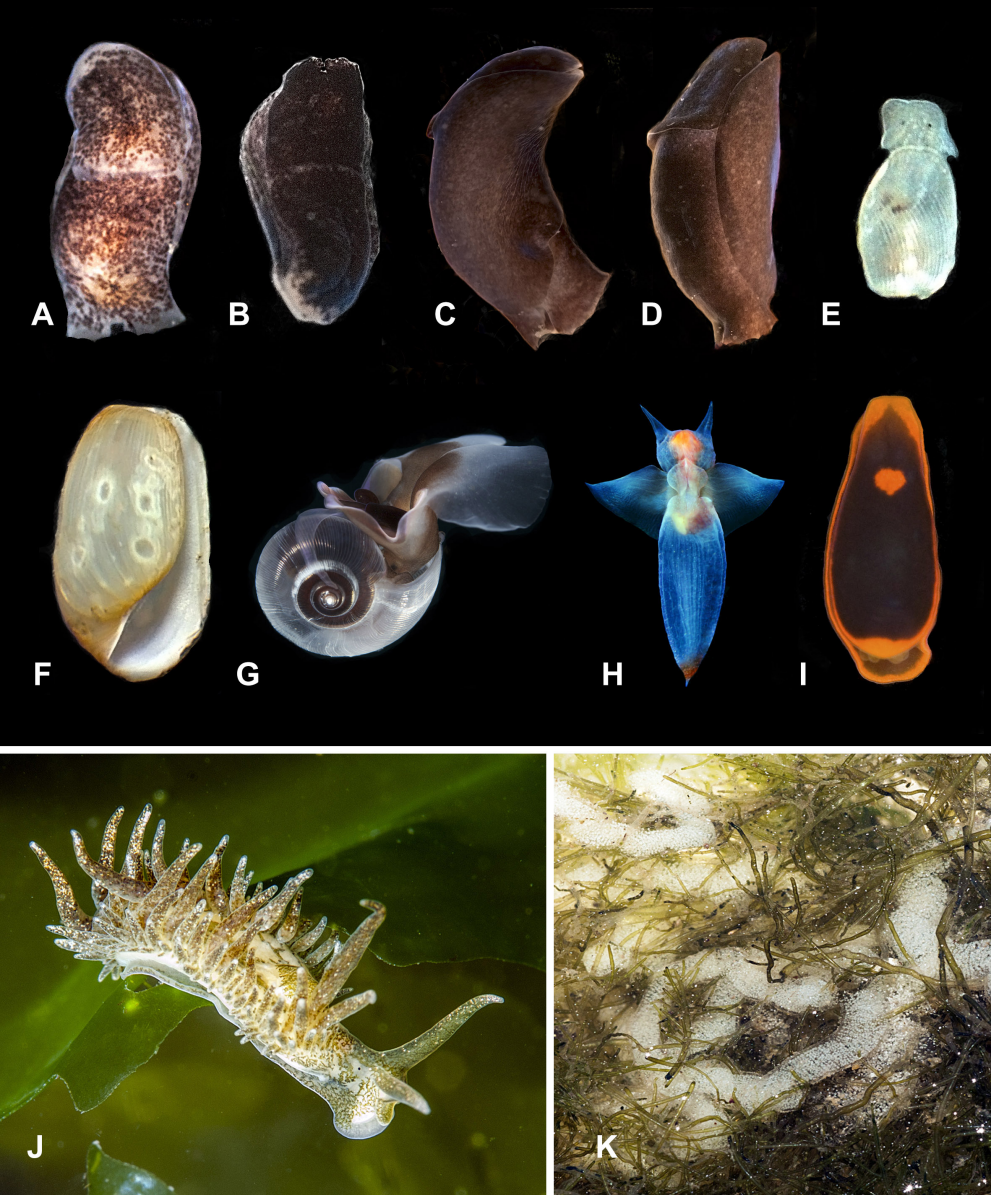
Heterobranchia of surveyed area. (A, B) *Melanochlamys yezoensis*, Rudnaya Bay. (C, D) *Melanochlamys* sp., Vladimir Bay. (E, F) *Retusa minima*, Kievka Bay. (G) *Limacina helicina*, Rudnaya Bay. (H) *Clione limacina*, Rudnaya Bay. (I) *Runcinida valentinae*, Senkina Shapka. (J) *Placida* cf. *babai*, Dva Brata. (K) egg mass of *P.* cf. *babai*, Nevelsk, Sakhallin.

**1. *Melanochlamys ezoensis* (Baba, 1957)** (Figs. 2A, 2B).

*Aglaja ezoensis*  Baba, 1957:8–14.

*Aglaja nana* Steinberg & Jones, 1960.

*Philinopsis giglioli*—Gulbin, 1990 (part.), non Tapparone-Canefri, 1874.

*Melanochlamys diomedea*—Chaban & Martynov, 1998 (part.); Chaban & Martynov, 2006 (part.); Gulbin & Chaban, 2009; Chaban & Martynov, 2013a (part.); Martynov & Korshunova, 2011 (part.), Yavnov, 2012 (part.), non Bergh, 1893.

**Material examined.** 2 specimens, Rudnaya Bay, 2m, 10 Oct 2015, A. Chichvarkhin leg.

**Identification.** Body length up to 7 mm. Background grey with dotted dark pigmentation.

**Ecology.** Occurs on the surface of sandy bottom, partially burrowed into sand.

**Distribution.** Japan, Korea, Russia (Primorye) (Martynov & Korshunova, 2011; Cooke et al., 2014).

**2. *Melanochlamys* sp.** (Figs. 2C and 2D)

? *Melanochlamys diomedea*—Yavnov, 2012 (part.), non Bergh, 1893.

**Material examined.** 7 specimens, Vladimir Bay, May 2014, 1–8 m, K. Dudka leg.

**Identification.** Superficially quite similar to sympatric *M. ezoensis* but adult individuals of *Melanochlamys* sp. approaching 14 mm body length are 3–4 times larger. These species also possess distinctive male reproductive system morphology (long penis, seminal bulb of approximately same size as the prostate) and DNA sequences (Breslau, Valdés & Chichvarkhin, in press).

**Ecology.** Occurs on the surface of sand bottom, partially immersed into sand mass. Probably feeds on sand-dwelling mollusks.

**Distribution.** Known from Vladimir Bay and South Korea. May occur in the continental shore of the Sea of Japan (Breslau, Valdés & Chichvarkhin, in press).

**Table:** 

**Superfamily Bulloidea Gray, 1827**
**Family Retusidae Thiele, 1925**
**Genus *Retusa* T. Brown, 1827**
**Type species** *Bulla obtusa* Montagu, 1803, by subsequent designation.

**3. *Retusa minima*** Yamakawa, 1911 (Figs. 2E and 2F)

Yamakawa, 1911:47, pl, 11, Figs. 21–24.

*Coleophysis (Sulcoretusa) minima* Habe, 1964; Kuroda, Habe & Oyama, 1971; Ito et al., 1986; Ito, 1990; Ishii, 1993.

*Retusa (Sulcoretusa) minima* Ito, 1978.

*Sulcoretusa minima* Higo & Goto, 1993; Higo, Callomon & Goto, 1999; Noseworthy, Lim & Choi, 2007.

*Cylichnina pertenuis*—Golikov & Scarlato, 1967 (part.), non Smith, 1875.

*Retusa (Cylichnina) succincta*—Minichev, 1971 (part.), non A. Adams, 1862.

*Tornatina truncata*—A. Adams, 1862; Kuroda & Habe, 1954, non *Bulla truncata* J. Adams, 1800.

**Material examined.** 2 specimens, Kievka Bay, 2 m, 1 Jul 2015, A. Chichvarkhin leg.

**Identification.** Shell-bearing mollusk. Differs from other similar Cephalaspidea with cylindrical shell shape and fine radial sculpture.

**Ecology.** Occurs on the surface of sand bottom, partially burrowed into sand.

**Distribution.** Previously known in Russia from Peter the Great Bay, also from Japan and Korea (Chaban & Chernyshev, 2009; Martynov & Korshunova, 2011).

**Table:** 

**Order Thecosomata Blainville, 1824**
**Superfamily Limacinoidea Gray, 1840**
**Family Limacinidae Gray, 1840**
**Genus *Limacina* Bosc, 1817**
**Type species** *Clio helicina* (Phipps, 1774), by monotypy.

**4. *Limacina helicina ochotensis*  Shkoldina, 1999a; Shkoldina, 1999b** (Figs. 2G)

Shkoldina, 1999a; Shkoldina, 1999b:299–305, Figs. 2 and 3.

**Material examined.** 6 specimens, Senkina Shapka pinnacle, 5 m, 5 May 2013, A. Chichvarkhin & A. Semenov leg.

**Identification.** Quite distinctive shelled planktonic species. Shell size ranges <1 to 8 mm.

**Ecology.** These planktonic mollusks appear in Spring and completely disappear at the end of May. Occur at the depths of 1–8 m at various sites. Rather rare. In summer time migrate to the lower depth. Feed on planktonic Diatoms collected with bubble-like mucous veil.

**Distribution.** This subspecies is known from southern Sea of Okhotsk and Primorye shore including Peter the Great Bay where reported very abundant at low depths in spring (Shkoldina, 1999a; Shkoldina, 1999b).

**Remark.** No polymorphism was detected in COI gene sequences of *L. helicina* from NW Sea of Japan is similar to those from N. Atlantic, N. Pacific, and Arctic: maximum p-distance between studied sequences (Table 1) of this species does not exceed 0.011 ± 0.004. This suggests identity of all these populations to a single species *L. helicina*.

**Table:** 

**Order Gymnosomata** **Blainville, 1824**
**Superfamily Clionoidea Rafinesque, 1815**
**Family Clionidae Rafinesque, 1815**
**Genus *Clione* Pallas, 1774**
**Type species** *Clio limacina* Phipps, 1774, by monotypy.

**5. *Clione limacina* (Phipps, 1774)** (Fig. 2H)

*Clio limacina*  Phipps, 1774:195–196.

*Clione dalli* Krause, 1855.

*Clione elegantissima*, Dall, 1871.

**Material examined.** 1 specimen, Klokovo Bay, 4 m, 11 May 2014, A. Chichvarkhin leg.

**Identification.** Very distinctive planktonic shell-less species. The form from the Sea of Japan differs by having a light caudal end of the body. Body size of adults 15–35 mm.

**Ecology.** These planktonic mollusks appear in spring and completely disappear at the end of May. Occur at the depths of 1–8 m at various sites. Not abundant. In summer time migrate to the lower depth. Obligated predator of planktonic *Limacina helicina*.

**Distribution.** Common in the Pacific, Atlantic and Arctic oceans (Martynov & Korshunova, 2011; Lebedev, Martynov & Korshunova, 2015).

**Table:** 

**Order Runcinacea Burn, 1963**
**Superfamily Runcinoidea H. Adams & A. Adams, 1854**
**Family Runcinidae H. Adams & A. Adams, 1854**
**Genus *Runcinida* Burn, 1963**
**Type species** *Runcina elioti* Baba, 1937a, by subsequent designation.

**6. *Runcinida valentinae*  Chernyshev, 2006** (Fig. 2I)

**Material examined.** 6 specimens, south of Rudnaya Bay, Senkina Shapka pinnacle, 18 m, 5 Jun 2013, A. Chichvarkhin leg.; 18 specimens, south of Rudnaya Bay, Senkina Shapka Pinnacle, 18–19 m, 15 May 2014, 18 m. A. Chichvarkhin leg.; 2 specimens, south of Rudnaya Bay, Senkina Shapka pinnacle, 16–19 m, 16 May 2015, A. Chichvarkhin leg.; 3 specimens Kievka Bay, 1.2 m, A. Chichvarkhin leg.

**Material examined.** Holotype: south of Rudnaya Bay, Senkina Shapka pinnacle, 16–19 m, 2 May 2016, A. Chichvarkhin leg.; Paratypes: 4 specimens, Rudnaya Bay, Senkina Shapka pinnacle, 15 May 2014, A. Chichvarkhin leg.

**Identification.** Body brown with violet tinge. Dorsum with bright orange rim and orange triangular or heart-shaped spot on third fore portion of the dorsum. Body length 2–6 mm.

Radula described and imaged in Chichvarkhin, Chichvarkhina & Chernyshev (2015).

**Ecology.** Occurs at the depths of 16–20 m on rocky substrates in Senkina Shapka pinnacle. In Kievka Bay lives at the depth of 0.5–3 m on calcareous red algae. Feeding presumably on benthic bacteria or protists, reproduction unknown.

**Distribution.** Originally described from Kunashir Island, referred as *Runcina elioti* from the northern Hokkaido (Nakano, 2004). Likely distributed along the Sea of Japan coast between Amur river mouth and Peter the Great Bay, probably in the Korean peninsula (Chernyshev, 2006; Chichvarkhin, Chichvarkhina & Chernyshev, 2015).

**Table:** 

**Order Sacoglossa Ihering, 1876**
**Superfamily Limapontioidea Gray, 1847**
**Family Limapontiidae Gray, 1847**
**Genus *Placida* Trinchese, 1876**
**Type species** *Calliopaea dendritica* Alder & Hancock, 1843, by monotypy.

**7. *Placida* cf. *babai* Ev. Marcus, 1982** (Figs. 2J and 2K)

*Placida babai* Ev. Marcus, 1982:25, Figs. 32 and 33.

*Placida* sp.–Fan et al., 2013.

*Placida dendritica*—Martynov, 1998b; Martynov, 2006; Martynov & Korshunova, 2011; Chernyshev, 2014, non Alder & Hancock, 1843.

*Placida dendritica* s. lato–Chaban & Martynov, 2013b.

*Hermaea dendritica*—(Baba, 1937).

*Placida dendritica*—Baba, 1955; Baba, 1959; Bleakney, 1989; Bleakney, 1990; Hamatani, 2000; Suzuki, 2000; Nakano, 2004; Trowbridge, Hirano & Hirano, 2008; Klochkova et al., 2010, non Alder & Hancock, 1843.

*Placida* sp.—Baba, 1986.

**Material examined.** 1 specimen, 5 Jun 2012 Dva Brata Rocks, 4 m, Chichvarkhin leg; 1 specimen, south of Oprichnik Bay, near Viking wreck site, on the rocks at sea surface level, ca. 100 m off shore, 6 Jun 2013, A. Chichvarkhin leg; 1 specimen, Vtoroy Is., Kievka Bay, 1 m, 3 Jul 2015, A. Chichvarkhin leg.

**Identification.** Body size reach 35 mm, usually smaller. background creamy white with green network of fine dendrites of digestive gland. Oral tentacles absent.

**Ecology.** in Russian waters, feeds on mainly on *Bryopsis* green algae. A report about feeding on *Ulva fenestrata* (Martynov & Korshunova, 2011) is likely due to a mistake.

**Distribution.** Confirmed from the Sea of Japan, Yellow Sea, and Pacific coast of Japan. Probably possesses wider distribution, which can be clarified after taxonomical problem solution concerned *P. babai* identity (Chichvarkhin et al., 2016c).

**Remarks.** The species occurring in the Sea of Japan are rather distinct in morphology and mitochondrial genes sequences from *P. dendritica* from the Atlantic. Therefore, this is a distinct species. However, it is difficult to assign proper taxonomical name for this species because of several unresolved taxonomical confusions (Chichvarkhin et al., 2016c).

**Table:** 

**Order Pleurobranchomorpha Pelseneer, 1906**
**Superfamily Pleurobranchoidea Gray, 1827**
**Family Pleurobranchidae Gray, 1827**
**Genus *Berthella* Blainville, 1824**
**Type species** *Bulla plumula* Montagu, 1803 (type by monotypy)

**Figure 3:**
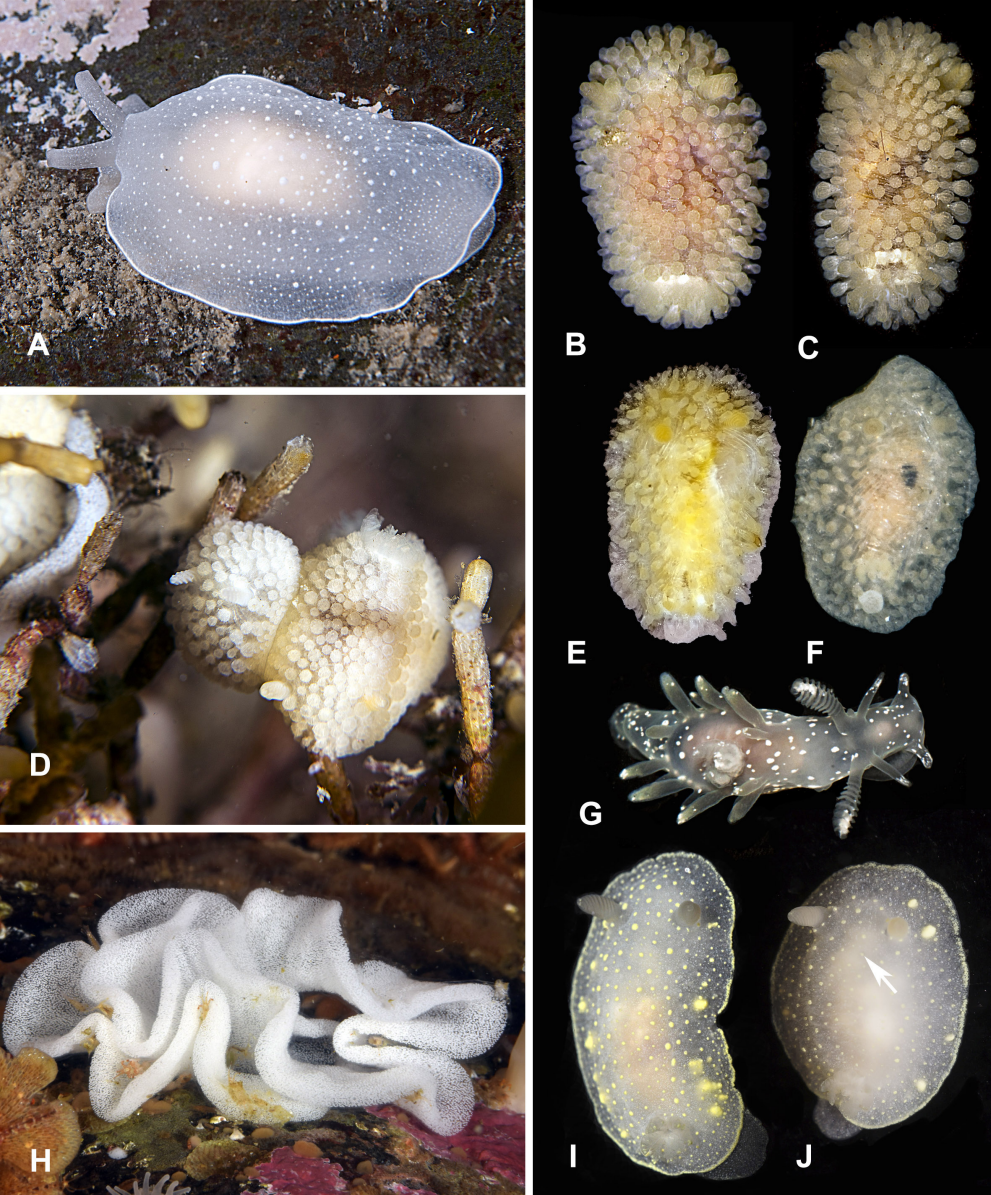
Heterobranchia of surveyed area. (A) *Berthella californica*, Senkina Shapka. (B–D) *Onchidoris muricata*, Senkina Shapka. (E, F) *Knoutsodonta jannae*, Kievka Bay. (G) *Ancula gibbosa*, Senkina Shapka. (H) *Berthella californica*, egg mass. (I, J) *Cadlina olgae*, Senkina Shapka.

**8. *Berthella californica* (Dall, 1900)** (Figs. 3A and 3H)

*Pleurobranchus californicus*  Dall, 1900:92–93.

*Pleurobranchus chacei* Burch, 1944.

*Pleurobranchus californicus denticulatus* MacFarland, 1966.

**Material examined.** 1 specimen, Cherniye Skaly Cape, 20 m, 5 Jun 2013, A. Chichvarkhin leg.; 1 specimen, Skaly Is., Kievka Bay, 7 m, 28 Jun 2015, A. Chichvarkhin leg.

**Identification.** White semi-translucent body with solid white dots and white rim around notum. No oral tentacles, tube-like rhinophores, head lobe wide. Body size to 80 mm, the specimens found in Primorye are max 45 mm. Gill covered by the right side of the notum.

**Ecology.** Occurs on the surface of rocky substrates at the depths of 10–30 m. Oviposits white egg ribbons onto lower side of the boulders. Feeding unknown.

**Distribution.** A common species known from California along North American and the Asian coast of Japan and Korea (Martynov & Korshunova, 2011).

**Table:** 

**Order Nudibranchia Cuvier, 1817**
**Superfamily Onchidoridoidea Gray, 1827**
**Family Onchidorididae Gray, 1827**
**Genus *Onchidoris* Blainville, 1816**
**Type species** *Onchidoris leachii* Blainville, 1816, by monotypy.

**9. *Onchidoris muricata* (Müller, 1776)** (Figs. 3B–3D)

*Doris muricata*  Müller, 1776:229.

**Material examined.** 2 specimens, Kievka Bay, 6 m, 1 Jul 2015, A. Chichvarkhin leg.; 12 specimens, Senkina Shapka pinnacle, 16–18 m, 15 May 2015, A. Chichvarkhin leg.

**Identification.** Color creamy white, size to 15 mm. Notum covered with bud-like (mushroom-like) tubercles.

**Ecology.** Feeds on a wide range of encrusting Bryozoans. In Senkina Shapka pinnacle feeds exclusively on different bush-like *Bugula articulata*. Occurs at the depth of 5–20 m. An ephemeral species that is abundant in May but totally disappeared in early autumn.

**Distribution.** Arctic and North Pacific species. Has been recently comfirmed from the Sea of Japan, far from its known distribution area (Chichvarkhin et al., 2016d).

**Genus *Knoutsodonta* Hallas & Gosliner, 2015**

**Type species** *Adalaria jannae* (Millen, 1987), by original designation

**10. *Knoutsodonta jannae* (Millen, 1987)** (Figs. 3E and 3F)

*Adalaria jannae*  Millen, 1987:2696–2702; Martynov, 2006; Martynov & Korshunova, 2011; Martynov, 2013.

? *Adalaria derjuguni* Volodchenko, 1941.

**Material examined.** 1 specimen, Kievka Bay, 5 m, 1 Jul 2015, A. Chichvarkhin leg.

**Identification.** Color creamy white to light brown, size to 12 mm. Notum covered with finger-like tubercles. White round gland behind the gills.

**Ecology.** Occurs at 1–15 m depth under stones and on rocks. Feeds on encrusting bryozoans.

**Distribution.** A common species known from California along North American and Asian coast to Peter the Great Bay. May occur in Japan and Korea (Martynov & Korshunova, 2011).

**Family Goniodorididae H. Adams & A. Adams, 1854**

**Genus *Ancula* Lovén, 1846**

**Type species** *Polycera cristata* Alder, 1841, by monotypy.

**11. *Ancula gibbosa* (Risso, 1818)** (Fig. 3H)

*Tritonia gibbosa*  Risso, 1818:

*Ancula pacifica* MacFarland, 1905.

*Polycera cristata* Alder, 1841.

**Material examined.** 2 specimens, north of Brynner Cape, Rudnaya Bay, 5–7 m, 10 May 2014, A. Chichvarkhin leg.

**Identification.** Body size to 15 mm, color white. Clearly distinguishable from other dorid nudibranchs by the long papillae near oral tentacles and around the rhinophores (Martynov & Korshunova, 2011).

**Ecology.** Occurs at 5–10 m. depth, feeds on bush-like bryozoans.

**Distribution.** North Pacific species.

**Superfamily Doridoidea Rafinesque, 1815**

**Family Cadlinidae Bergh, 1891**

**Genus *Cadlina*  Bergh, 1879**

**Type species** *Doris laevis* Linnaeus, 1767, by monotypy.

**Figure 4:**
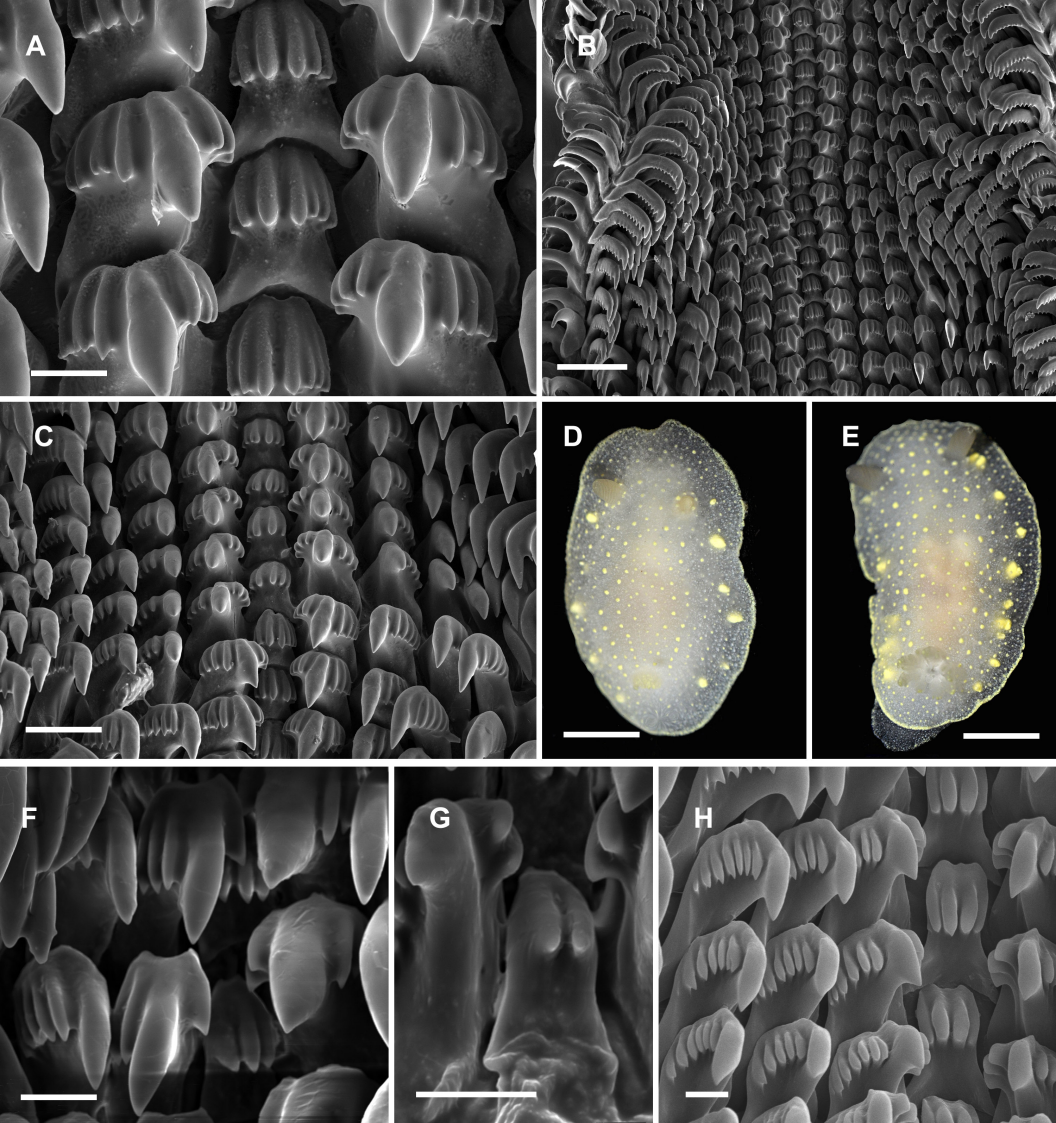
Radular and extrenal morphology of *Cadlina* spp. *Cadlina olgae*: (A) rachidian and first marginal teeth of 29th and 30th rows, scale 10 mkm; (B) overwiew of radula rows, scale 50 mkm; (C) 37–43th rows; (D) holotype, scale 3 mm; (E) paratype, scale 3 mm. *Cadlina laevis* (White Sea): (F) rachidian and central lateral teeth, scale 10 mkm; (G) rachidian tooth of 50th row, scale 10 mkm; *Cadlina* sp.1: (H) rachidian and first marginal teeth of 29th and 30th rows, scale 10 mkm.

**12. *Cadlina olgae* sp. nov.** (Figs. 3I, 3J and 4A–4E)

urn:lsid:zoobank.org:act:758A5BFF-FDB9-4E19-8D0D-D054358ACE6F

*Cadlina laevis*—Martynov, 2006 (part.); Martynov & Korshunova, 2011 (part.), non Linnaeus, 1767.

? *Cadlina* spp.—(Martynov, 2013) (part.).

**Type material.** Holotype: MIMB 33105 Senkina Shapka pinnacle, south of Rudnaya Bay, 16 m, 10 Oct 2015, O. Krutichenko leg.; Paratype: MIMB33106 Senkina Shapka pinnacle, south of Rudnaya Bay, 14 m, 6 May 2013, T. Antonkhina leg.

**Material examined.** 1 specimen, Senkina Shapka pinnacle, south of Rudnaya Bay, 16 m, 10 Oct 2015, O. Krutichenko leg.; Dva Brata Rocks, south of Rudnaya Bay, 4 m, 16 May 2014, A. Chichvarkhin leg.

**Diagnosis.** White semi-translucent oval shaped body with solid yellow dots, rather large yellow glands near the edge of mantle, and yellow rim formed by numerous tiny dots around notum. Oral tentacles short, triangular, folded at apex, rhinophores lamellar. Rachidian teeth with 2 bigger central and 4–6 smaller lateral denticles. Inner lateral teeth with equal number of denticles on both sides. Body size to 25 mm.

**Description.** Body shape oval, rounded in juvenile specimens, lengths to 25 mm (14 mm in holotype, 11 mm in paratype) in fully extended living specimens (Figs. 4D and 4E). Body with uniformly white semi-translucent background, uniformly covered with small yellow dots on elevated tubercles. 4–10 larger yellow sub-epidermal glands along each side of mantle; edge of notum and foot covered with numerous tiny dots forming yellow rim, which looks solid without magnification (but less intense than in *C. luteomarginata* MacFarland, 1966). Notum moderately wide, wider than foot, contains no spicules. Rhinophores with 8–10 lamellae with few yellow dots on top. Oral tentacles very short, triangular, folded distally. Gills in holotype with five branchial leaves, with yellow pigment on tips. Radula (Fig. 4) of 55–60 rows, in 30th row 12.1.1.1.12. Rachidian tooth with two central larger central denticles and 2–3 smaller lateral denticles (Fig. 4A). First lateral teeth with bigger central denticle and four smaller denticles on both sides. The other lateral teeth are similar, with 4–5 outer denticles and no inner denticles (Fig. 4C).

Ampulla wide, long and convoluted in two folds. Prostate long, tubular with 1–2 loops, vas deferens very narrow with one loop, it expands in wider muscular ejaculatory portion. Penis narrow, bears an armature of very fine spines. Vagina wide and short, branched into a duct that connects seminal receptacle and uterine duct. Uterine duct is long, not shorter than bursa copulatrix. Seminal receptacle almost spherical, slightly smaller than oval bursa copulatrix. No vagina extension near the entrance into copulatory bursa

**Etymology.** After my wife and colleague Olga Chichvarkhina.

**Ecology.** Occurs at various depths on rocky substrates, feeding unknown.

**Distribution.** Probably has wider distribution in the Sea of Japan.

**Remarks**. This species differs from *Cadlina* sp. (Martynov, 1999) with larger rachidians and fewer denticles in lateral teeth. Central denticles in the rachidian tooth of *C. olgae* are never split in 2–3 secondary denticles. The invalid (unpublished) species “*Cadlina potini*’ referred by Martynov (1999) is more similar to *C. olgae* but possesses 6 outer denticles in first lateral teeth (four in *C. olgae*), the other lateral teeth possess 15 lateral denticles (4–5 in *C. olgae*). Both these forms referred by Martynov, the radula possesses more rows with more teeth in each row. In *C. laevis* (Linnaeus, 1767), rachidian teeth possess up to six equal denticles (unequal in *C. olgae*) (Thompson & Brown, 1984). Examined specimens of *C. laevis* form the White Sea possess rachidian tooth with 2–4 poorly developed smooth denticles; first lateral tooth is crowned with three denticles on inner side and 5–7 denticles on the outer side (Figs. 4F and 4G), similar pattern is observed in *C*. sp.2 from Bering Sea (Fig. 4H) (four denticles on both sides in *C. olgae*). *C. japonica* Baba, 1937 clearly differs from *C. olgae* with: brownish pigment on the mantle, intense yellow pigmentation of gills, small hook-shaped rachidian tooth divided in two lobe-like denticles, and presence of small outermost lateral teeth (Baba, 1937b). *C. luteomarginata* MacFarland, 1966 differs from *C. olgae* with solid yellow rim around the mantle, more intense pigmentation on the tubercles, hook-shaped rachidian tooth with four small denticles, larger central denticle on all lateral teeth, and 7–8 very small denticles on all lateral teeth (Rudman, 2001; Johnson, 2001). Reproductive system is typical for Far Eastern *C. laevis*-group species described in Martynov (1999): it possesses rather polymorphic prostate and vas deferens containing one to five loops, thus they unlikely can be served as species-specific traits. Female reproductive system is similar to Martynov’s (1999) “*C. potini*” (in *C. olgae* holotype is identical with Fig. 83 in this work) with no vaginal duct extension near bursa copulatrix entrance. I suppose, Martynov (1999) studied *C. olgae* but he mixed it with one or more species reporting radula/reproductive combinations, that do not fully coincide with my specimens. Thus thorough study of morphological variation in *Cadlina* needed to shed light onto the systematics of this genus in the northwestern Sea of Japan.

**Figure 5:**
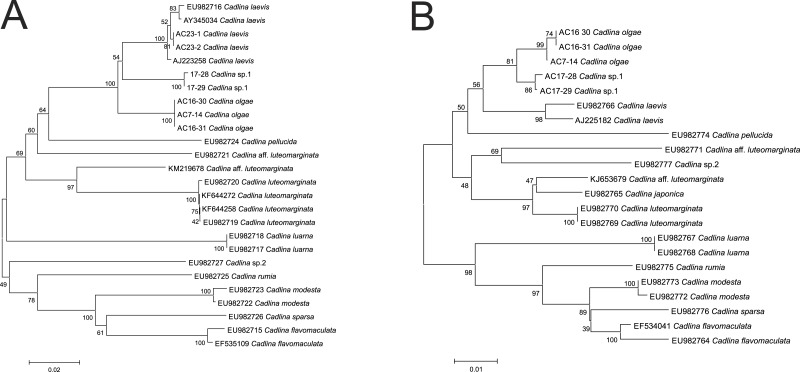
Cladistic species dilimitation in the genus *Cadlina:* Neibour Joining tree. Bootstrap support (1,000 pseudoreplicates) shown at the internodes. (A) COI. (B) 16S.

Molecular COI sequences suggest an evidence that *Cadlina olgae* is a member of cryptic species complex referred as *C. laevis*, which includes at least *C. olgae, C. laevis*, an undescribed species candidate from Bering Sea, and *C. luteomarginata* with at least two sister species (Fig. 5). Although the p-distance between these species is relatively low, lowered level of divergence is a characteristic for sibling species that descent during Pleistocene glaciations (Breslau, Valdés & Chichvarkhin, in press; Lindsay et al., 2016; Klienberger et al., 2016; Hallas, Simison & Gosliner, 2016). Likely, this phenomenon also occurs in amphiboreal species with direct development, e.g., *Cadlina* (Thompson, 1967) whose speciation took place during recent dispersal from a refugia.

The resulted number of species identified in ABGD analysis of COI and 16S. Using uncorrected distance matrices, the COI sequences showed a major barcode gap between a priori genetic distance thresholds of 0.01 and 0.036 in COI (0.01 and 0.013 in 16S). Using a value of *P* between this range (0.01 for both markers), the same 13 species were identified, and assignment of individuals to the species matched the NJ tree topology (Fig. 5). Importantly, however, the species identified are not polyphyletic. A series of species-specific diagnostic indels were found in the 16S after positon #240 (in *C. laevis* sequence): there is no insert in the *C. olgae*, while a six-base TTTTTA insert is present in *C. laevis* sequence, and eight-base insertion ATTTTTTA in *C*. sp. 1 (Table 2). These indels are likely a conservative trait in *Cadlina* species because *C. luarna* and *C. rumia* do not possess an insert as does *C. olgae*, while three species (*C. japonica, C. luteomarginata, C.* aff. *luteomarginata*) possess a four-base insert TTT(C)A, three others possess one Thymidine insert (*C. flavomacualta, C. modesta*, and *C. sparsa*), *C. pellucida* possesses a four-base TTTA insert, and *C*. sp.2 possesses an insert of seven bases TTTTAAA. I suppose this pattern has high phylogenetic weight, hence it is capable to adequately detect closely-related sibling species.

**Table 2:** Partial 16S sequences of the species in the genus *Cadlina* (positions #221-255 in *C. laevis*) with barcoding indels after position #240.

*laevis* Norway	**G** CTTTACTAA **-G** TT **G** AAAAT**–**TTTTTA**–**TTTTCAA **G** A
*laevis* Sweden	**G** CTTTACTAAA **G** TT **G** AAAAT**–**TTTTTA**–**TTTTTAA **G** A
*olgae*	**G** CTTTACTAAA **G** TT **G** AAATT**———-**TTTTCAA **G** T
sp.1 Bering Sea	**G** CTTTACTAAA **G** TT **G** AAATTATTTTTTA**–**TTTTCAA **G** T
sp.2 S. Africa	**G** CTTT **G** CTAAA **G** TTAA **G** AAT**–**TTTTAAATTCTT **G** AAT
*japonica*	**G** CTTTACTAAAATT **G** A **G** A **G** T**–**TTCTA**–**TTCTTAA **G** T
*luteomarginata*	**G** CTTCACTAAA **G** TT **G** A **G** AAT**–**TTTTA**–**TTCTTAA **G** T
aff. *luteomarginta*	**G** CTTTACTAAA **G** TT **G** A **G** AAT**–**TTTTA**–**TTCTTAA **G** T
*luarna*	**G** TTTTACTAAAATTAAATT **G———-** TTTTTAA **G** T
*pellucida*	**G** CTTTACTAAA **G** TT **G** AAAAT**—-**TTTA**–**TTTTTAAAA
*rumia*	**G** CTTTACTAAA **G** TT **G** AATCT**———-**TTTTTAA **G** T
*flavomaculata*	**G** CTTTACTAAAATT **G** AATTCT**——–**TTTTAA **G** T
*modesta*	**G** CTTTACTAAAATT **G** AATTCT**——–**TTTTAA **G** T
*sparsa*	**G** CTTTACTAAAATT **G** AATTCT**——–**TTTTAA **G** T

**Table:** 

**Family Discodorididae Bergh, 1891**
**Genus *Diaulula* Bergh, 1878**
**Type species** *Diaulula sandiegensis* Cooper, J.G., 1863, by monotypy.

**Figure 6:**
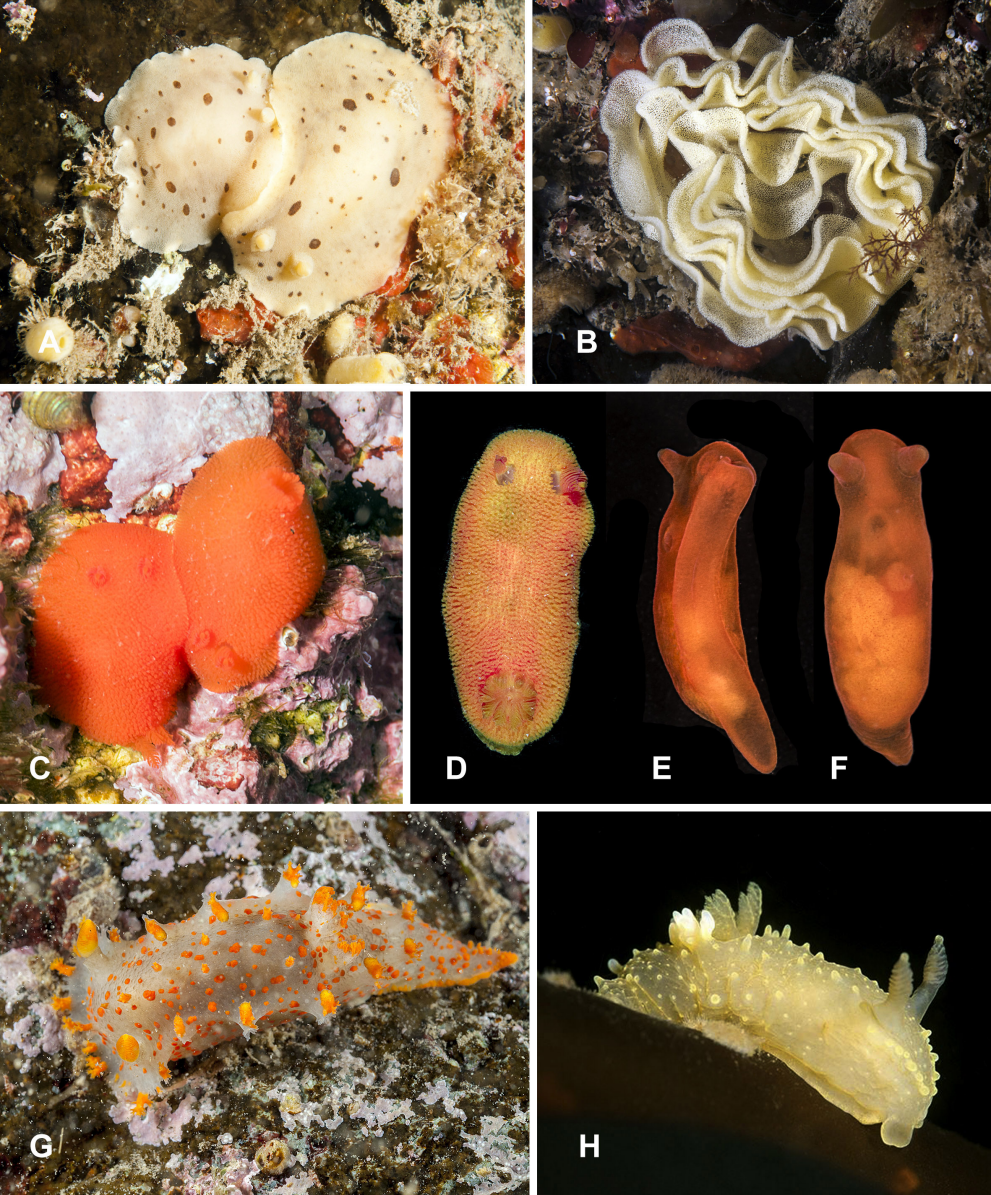
Heterobranchia of surveyed area. (A) *Diaulula odonoghuei*, Brynner Cape. (B) *D. odonoghuei* egg mass.(C, D) *Rostanga alisae*, Kievka Bay. (E, F) *Vayssierea elegans*, Kievka Bay. (G) *Triopha catalinae*, Oprichnik Bay. (H) *Palio dubia*, Klokovo Bay.

**13. *Diaulula odonoghuei*  Steinberg, 1963** (Figs. 6A and 6B)

Steinberg, 1963:63–67.

*Peltodoris mauritana*—Baba, 1935a; Baba, 1935b, non Bergh, 1889.

*Archidoris tuberculata*—Volodchenko, 1941; Volodchenko in Ushakov, 1953 (non Cuvier, 1804).

*Doris echinata*—O’Donoghue, 1922 (non Lovén, 1846).

*Doridigitata maculata*—O’Donoghue, 1926 (non Garstang, 1896).

*Doris odonoghuei*—Behrens & Valdés, 2001.

*Diaulula sandiegensis*—Behrens, 1980 (part.); Martynov, 2006; Martynov & Korshunova, 2011; Martynov, 2013 non *Doris (Actinocyclus?) sandiegensis* Cooper, 1863.

**Material examined.** 1 specimen, Rudnaya Bay, Brynner Cape, 5–6 m, 10 May 2014, A. Chichvarkhin leg; 2 specimens, Senkina Shapka pinnacle, 12–16 m, 12 May 2014, A. Chichvarkhin leg; 1 specimen Dva Brata Rocks, 5–6 m, 6 Jun 2013, leg. A. Chichvarkhin; 1 specimen, Kievka Bay, 5–6 m, 29 Jun 2015, A. Chichvarkhin leg.

**Identification.** Creamy-yellowish body color with dork brown large spots. Notum covered with numerous fine caryophillidiae.

**Ecology.** Occurs at the depths of 1–30 m, feeds on *Adocia cinerea* and *Haliclona permolis* sponges.

**Distribution.** South Korea, Japan, Russian Pacific, Kommander’s Islands, to Alaska and Northern California (Lindsay et al., 2016).

**Remark.** This species had been referred to *D. sandiegensis* (Cooper, 1863) that occurs in Pacific coast of North America, but our recent study has confirmed distinctiveness of these species (Lindsay et al., 2016).

**Genus** ***Rostanga*  Bergh, 1879**

**Type species** *Doris coccinea* Forbes, 1848, by monotypy.

**14. *Rostanga alisae*  Martynov, 2003** (Figs. 6C and 6D)

Martynov, 2003:142–146, Figs. 1–3.

**Material examined.** 2 specimens, Kievka Bay, 2 m, 29 Jun 2015, A. Chichvarkhin leg.

**Identification.** Very distinctive intense orange-red colored dorid nudibranch with characteristic rosette-like rhinophores formed with vertical lamellae, notum covered with numerous small caryophyllidiae. Body size to 16 mm.

**Ecology.** Occurs at 0–10 m depth, feeds on *Ophlitaspongia pennata* sponge.

**Distribution.** Northern continental shore of the Sea of Japan (Martynov & Korshunova, 2011).

**Table:** 

**Superfamily Polyceroidea Alder & Hancock, 1845**
**Family Okadaiidae Baba, 1930**
**Genus *Vayssierea* Risbec, 1928**
**Type species** *Vayssierea caledonica* Risbec, 1928, by original designation.

**15. *Vayssierea elegans* (Baba, 1930)** (Figs. 6E and 6F)

*Okadaia elegans*  Baba, 1930:48–50, pl. 2, Figs. 11–14.

*Okadaia tecticardia* Slavoshevskaya, 1971.

**Material examined.** Three specimens, Kievka Bay, 1–2 m, 1–5 Jul 2015, A. Chichvarkhin leg.

**Identification.** Small red-colored mollusk with elongate body. Body smooth: gill, tentacles or papillae on the notum are absent. Body size to 6 mm.

**Ecology.** Occurs at shallow depth of 0.1–2 m under rocks or on algae. Feeds on Spirorbidae tube worms.

**Distribution.** Known from Kievka and Peter the Great Bays in Russia, also from Japan (Martynov & Korshunova, 2011).

**Table:** 

**Family Polyceridae Alder & Hancock, 1845**
**Genus *Triopha* Bergh, 1880**
**Type species** *Triopa carpenteri* *S*tearns, 1873, by monotypy.

**16. *Triopha catalinae* (Cooper, 1863)** (Fig. 6G)

*Triopa catalinae*  Cooper, 1863:59.

*Triopa carpenteri* Stearns, 1873.

*Triopha modesta* Bergh, 1880.

*Triopha scrippsiana* Cockerell, 1915.

*Triopha elioti* O’Donoghue, 1921.

*Triopa pacifica* Volodchenko, 1941.

**Material examined.** 2 specimens, Tretya Langou, 14 m, 12 May 2014, A. Chichvarkhin leg; 2 specimens, 8 m, Dva Brata Rocks, 13 May 2014, A. Chichvarkhin leg; 1 specimen, Kievka Bay, 7 m, 29 Jun 2015, A. Chichvarkhin leg.; 2 specimens, Senkina Shapka Pinnacle, 17 m, 2 Jun 2016, A. Chichvarkhin leg.

**Identification.** Background body color varies bright white to light grey with orange pigment on the gills tips and papillae located on notum edge, darker orange colored tubercles scattered on notum. Body size to 15 cm.

**Ecology.** Occurs at 1–30 m depth, feeds on various bryozoans (Martynov, 1999).

**Distribution** A common species known from California along North American and Asian coast to Japan and Korea (Martynov & Korshunova, 2011).

**Genus *Palio* Gray, 1857**

**Type species** *Polycera ocellata* Alder & Hancock, 1842, by monotypy.

**17. *Palio dubia* (Sars, 1829)** (Fig. 6H)

*Palio dubia*—Martynov, 2006; Martynov & Korshunova, 2011.

*Palio* sp.—Martynov, 2013.

**Material examined.** 1 specimen, Senkina Shapka pinnacle, 5 May 2013, 16 m, A. Chichvarkhin leg.

**Identification.** Background color grey, greenish-grey with numerous light tubercles. Rhinophores lamellated, larger whitish tubercles behind the gills. Size to 15 mm.

**Ecology.** Occurs on 5–20 m depth, feeds on encrusting bryozoans.

**Distribution.** North Atlantic, White Sea, Barents Sea, North Pacific (Martynov & Korshunova, 2011).

**Table:** 

**Superfamily Tritonioidea Lamarck, 1809**
**Family Dendronotidae Allman, 1845**
**Genus *Dendronotus* Alder & Hancock, 1845**
**Type species** *Doris arborescens* Müller, 1776, by monotypy.

**Figure 7:**
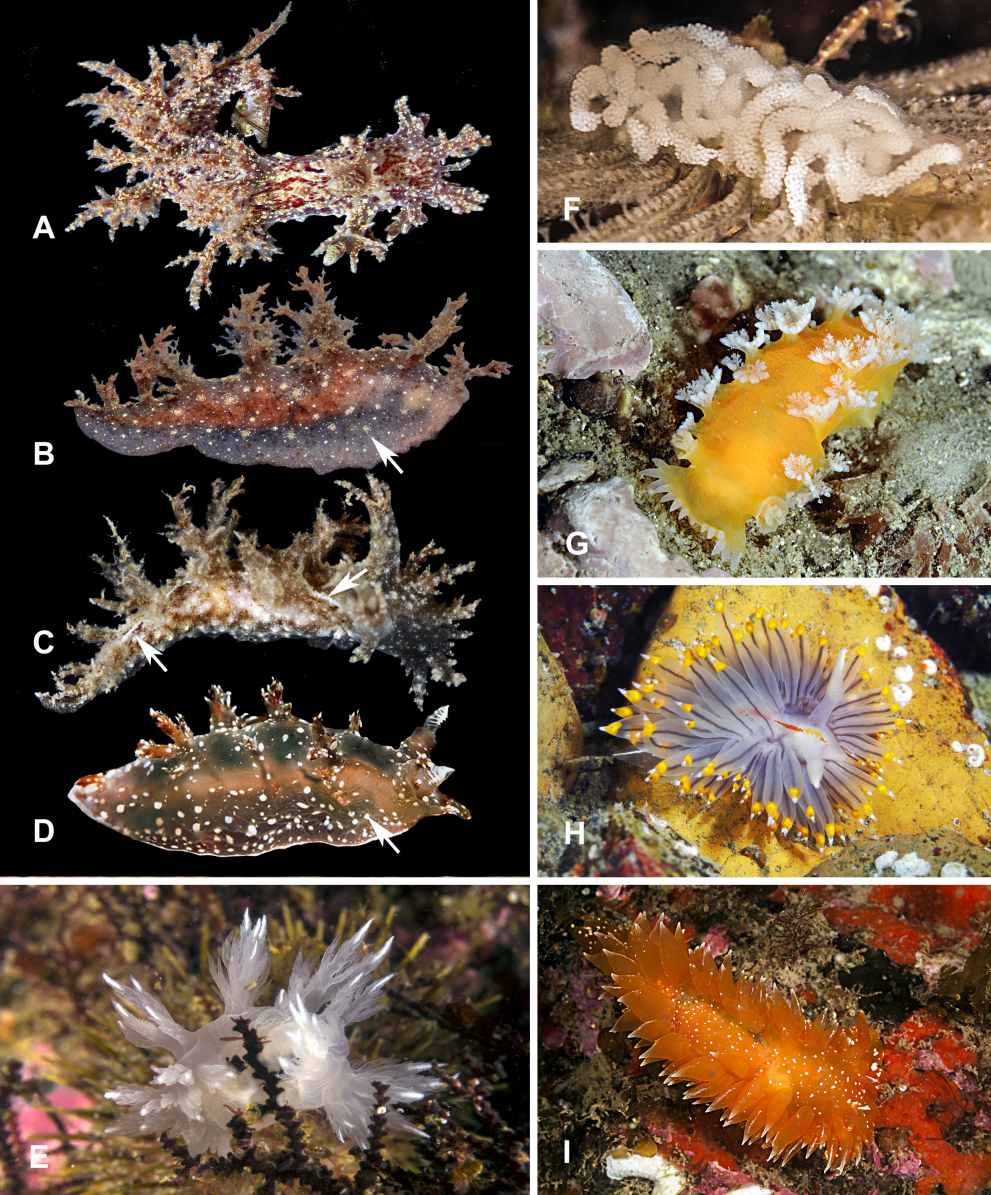
Heterobranchia of surveyed area. (A) *Dendronotus kamchaticus*, Rudnaya Bay. (B) *D. frondosus*, Rudnaya Bay. (C) *D. dudkai*, Rudnaya Bay. (D) *D. albopunctatus*, Rudnaya Bay. (E) *D. dallii*, Avacha Bay. (F) *D. kamchaticus* egg mass. (G) *Tritonia tetraquetra*, Nevelsk, Sakhalin (H) *Janolus fuscus*, Rudnaya Bay. (I) *Dirona pellucida*, Rudnaya Bay.

**18. *Dendronotus kamchaticus*  Ekimova et al., 2015** (Figs. 7A and 7F)

*Dendronotus frondosus*—Martynov, 2006; Ekimova et al., 2015; Martynov & Korshunova, 2011:152-155 (part.), non Ascanius, 1774.

? *Dendronotus robustus*—Yavnov, not Verrill, 1870

? *Dendronotus primorjensis* Martynov, Korshunova & Sanamyan, 2015.

**Material examined.** 2 specimens, Rudnaya Bay, 8 May 2013, A. Chichvarkhin leg; 1 specimen, Rudnaya Bay, 10 Oct 2015, A. Chichvarkhin leg.

**Identification**. Oral veil with 4–6 lip papillae and branched appendages. Primary stalks of veil appendages tall and slender, giving rise to numerous secondary branches with short tertiary branches. Rhinophoral sheath divide into 5–6 crown papillae that about same length. Lateral papillae (about one-third or one-half of sheath length) branches off sheath base and expanded with secondary branches. Rhinophores bear 14–20 lamellae. Background color is transparent white, with complex pattern of light, dark, and red-brown spots and stripes. On dorsal side spots and stripes merge and form characteristic striped pattern. Lateral sides of body devoid of stripes but covered with brown spots. Size to 25 mm.

**Ecology.** Occurs at 10–20 m depth on cnidarians.

**Distribution.** Described from Kamchatka, recently found in Rudnaya and Peter the Great Bays. Probably possess wide distribution along Far Eastern shore (Ekimova et al., 2016).

**19. *Dendronotus frondosus*  Ascanius, 1774** (Fig. 7B)

*Amphitrite frondosa* Ascanius, 1774: 155, pl. 2, Fig. 2.

*Dendronotus primorjensis* Martynov, Korshunova & Sanamyan, 2015.

? *Dendronotus frondosus* s.l.—Chernyshev, 2014.

**Material examined.** 1 specimen, Rudnaya Bay, 10 Oct 2015, A. Chichvarkhin leg.

**Identification**. Body slim elongate laterally compressed with 4–10 pairs of branched papillae. Oral veil with 10–14 short lip papillae and 4–5 secondary branched appendages. Rhinophoral sheaths with long stalk and five crown appendages. Lateral papillae moderate in size with small secondary branches. Light to dark brown body with opaque golden groups of dots. Size to 20 mm.

**Ecology**. Occurs at 1–20 m depth on cnidarians, mainly on *Obelia* sp.

**Distribution**. North Atlantic, Barents Sea, White Sea, the northern part of the Sea of Japan (Ekimova et al., 2016).

**20. *Dendronotus dudkai*  Ekimova et al., 2016** (Fig. 7C)

? *Dendronotus frondosus*s.l.–Chernyshev, 2014:93.

? *Dendronotus primorjensis* Martynov, Korshunova & Sanamyan, 2015.

**Material examined.** 1 specimen, Rudnaya Bay, 10 June 2012, A. Chichvarkhin leg.; 5 specimens, Rudnaya Bay, 8 Oct 2013, A. Chichvarkhin leg.

**Identification**. Superficially similar to sympatric *D. frondosus* but possess perl-white stripes along the dorsal side. Oral veil small with 6–12 large, secondary branched cerata. Muscular lips with 5–10 short lip papillae. Rhinophoral sheaths with long stalk and 4–5 crown secondary branched appendages. Lateral papillae moderate in size with small secondary branches. Rhinophores with 8–10 lamellae. 6–8 pairs of highly branched dorsolateral processes, size and degree of branching decrease towards the tail. Size to 20 mm.

**Ecology**. Occurs at 10–20 m depth on *Obelia* cnidarians.

**Distribution.** This species has been detected just recently. It’s confirmed distribution is two locations in Peter the Great Bay, and Rudnaya Bay, but may have wider distribution.

**Remark.** Recently, *Dendronotus primorjensis* Martynov, Korshunova and Sanamyan, 2015 has been described from Peter the Great Bay where at least three *Dendronotus* species occur. The description of the external morphology is quite brief and literally constitutes a redescription of *D. kamchaticus* because of the absence of white pigment agglomerations described for *D. primorjensis* is a characteristic of *D. kamchaticus*. However, described radula conforms to diagnosis of all species in the *D. frondosus* species complex. The illustrated holotype cannot be distinguished from *D. kamchaticus*, thus, *D. primorjensis* is probably a synonym of *D. kamchaticus*. The location of the type specimens of *D. primorjensis* is unknown: probably they do not exist because of their unavailability in referred collection, while the authors refuse providing them for examination. Also, the authors cannot provide or publish *D. primorjensis* nucleotide sequences that they refer as “distinct from the other *Dendronotus* species.” Therefore, we suggest considering *D. primorjensis* as *nomen nudum* or a synonym of a species of *D. kamchaticus* that is likely occurs at the type locality of *D. primorjensis* (Ekimova et al., 2016).

**21. *Dendronotus* cf. *albopunctatus*  Robilliard, 1972** (Fig. 7D)

Robilliard, 1972:421–432.

**Material examined.** Several specimens, about 2 cm long were photographed by Andrei Shpatak and Andrei Nekrasov in Rudnaya Bay area.

**Identification.** Wide body with short papillae and solid white dots on small tubercles.

**Ecology.** Unknown.

**Distribution.** The species is known from northeastern Pacific only, never been confirmed from Asian coast.

**22. *Dendronotus dalli*  Bergh, 1879** (Fig. 7E)

(Bergh, 1879):150, pl. 1, Fig. 21, pl. 2, Figs. 9–12, pl. 3, Figs. 2–6.

*Dendronotus elegans*—Verrill, 1880.

**Material examined.** 1 specimen, 4 cm long was imaged by Andrei Shpatak in June, 2013 at Dva Brata Rocks (http://shpatak.livejournal.com/175711.html).

**Identification.** Color varies: white, yellow, creamy to dark orange. Usually six pairs of papillae with solid white pigmented tips.

**Ecology.** Occurs at 5 m deeper depths. Feeds on hydroids.

**Distribution.** A common species known from California along North American and Asian coast to Sakhalin, Japan and Primorye.

**Table:** 

**Family Tritoniidae Lamarck, 1809**
**Genus *Tritonia* Cuvier, 1798**
**Type species** *Tritonia hombergii* Cuvier, 1803, by subsequent designation.

**23. *Tritonia tetraquetra* (Pallas, 1788)** (Fig. 7G)

*Limax tetraquetra*  Pallas, 1788, non *Tochuina tetraquetra*  Bergh, 1879.

*Tritonia diomedea* Bergh, 1894.

*Tritonia primorjensis* Minichev, 1971.

**Material examined.** 1 specimen, Nevelsk, Sakhalin Is, 10 m, 22 Aug 2014, A. Chichvarkhin leg.; 1 specimen, Kholmsk, Sakhalin Is, 7 m, 26 Aug 2014, A. Chichvarkhin leg.

**Identification.** Very distinctive orange-colored bode with white plumage-like papillae. Body size usually 20–50 mm but may grow to 300 mm.

**Ecology.** Occurs 1–2 m and deeper.

**Distribution.** Rare along continental shore of the Sea of Japan (Minichev, 1971). Very common on is adjacent Sakhalin shore. Occurs also in all Russian Pacific seas and along American coast to California (Martynov & Korshunova, 2011).

**Table:** 

**Unassigned Cladobranchia**
**Family Proctonotidae Gray, 1853**
**Genus** ***Janolus* Bergh, 1884**
**Type species** *Janolus australis* Bergh, 1884, by monotypy.

**24. *Janolus fuscus* O’Donoghue, 1924** (Fig. 7H)

O’Donoghue, 1924:1–33.

**Material examined.** 1 specimen, Senkina Shapka pinnacle, 5 May 2013, 16 m, T. Antokhina leg.; 1 specimen, Senkina Shapka pinnacle, 16 m, 14 May 2014, A. Chichvarkhin leg.; 1 specimen, Senkina Shapka pinnacle, 18 m, 15 May 2015, A. Chichvarkhin leg.

**Identification.** Distinctive species with numerous long semi-translucent white body and papillae with dark digestive gland inside and yellow circles below solid white tips. Brown line along dorsum. Size to 35 mm.

**Ecology.** Associated with various bryozoan hosts. In Senkina Shapka, feeds on *Bugula articulata* colonies only at the depths of 16–19 m.

**Distribution.** From Baja California to Alaska in America, also in Japan and Korea. In Russia, known from Senkina Shapka site only (Chichvarkhin et al., 2016; Behrens & Hermosillo, 2005).

**Table:** 

**Family Dironidae Eliot, 1910**
**Genus *Dirona* MacFarland, 1905**
**Type species** *Dirona picta* MacFarland, 1905, by subsequent designation.

**25. *Dirona pellucida*  Volodchenko, 1941** (Fig. 7I)

Volodchenko, 1941:56, 65, pl. 1, Fig. 6, pl. 2, Fig. 6.

*Dirona akkeshiensis* Baba, 1957.

*Dirona aurantia* Hurst, 1966.

*Dirona albolineata*—Volodchenko, 1941, non Eliot in Cockerell & Eliot ex MacFarland, 1905.

*Dirona picta*—Volodchenko, 1941, non Eliot in Cockerell & Eliot ex MacFarland, 1905.

**Material examined.** 2 specimens, Rudnaya Bay, Brynnera Cape, 5 m, 6 May 2013, A. Chichvarkhin leg.; 3 specimens, Senkina Shapka pinnacle, 15–18 m, 6 May 2013, A. Chichvarkhin leg.; 2 specimens, Dva Brata rocks, 5 m, 6 May 2013, A. Chichvarkhin leg.; 2 specimens, Senkina Shapka pinnacle, 16 m, 15 May 2014, A. Chichvarkhin leg.; 1 specimen, Dva Brata rocks, 7 m, 13 May 2014, A. Chichvarkhin leg.; 1 specimen, Senkina Shapka pinnacle, 14 m, 15 May 2015, A. Chichvarkhin leg.; 1 specimen, Senkina Shapka pinnacle, 17 m, 10 Oct 2015, A. Chichvarkhin leg.; 4 specimens, Senkina Shapka pinnacle, 15–20 m, 2 Jun 2015, A. Chichvarkhin leg.

**Identification.** Semi-translucent pale yellow to intensive orange body and flattened papillae. White dots scattered across the body, the tips of papillae white. No white rim around foot. Size to 150 mm.

**Ecology.** Occurs on rocky substrates at various depths. Feeding unknown.

**Distribution.** A common species known from California along North American and Asian coast to Japan and Korea (Martynov & Korshunova, 2011).

**Table:** 

**Superfamily Flabellinoidea Bergh, 1889**
**Family Flabellinidae Bergh, 1889**
**Genus *Flabellina* Gray, 1833**
**Type species** *Doris affinis* Gmelin, 1791, by monotypy.

**Figure 8:**
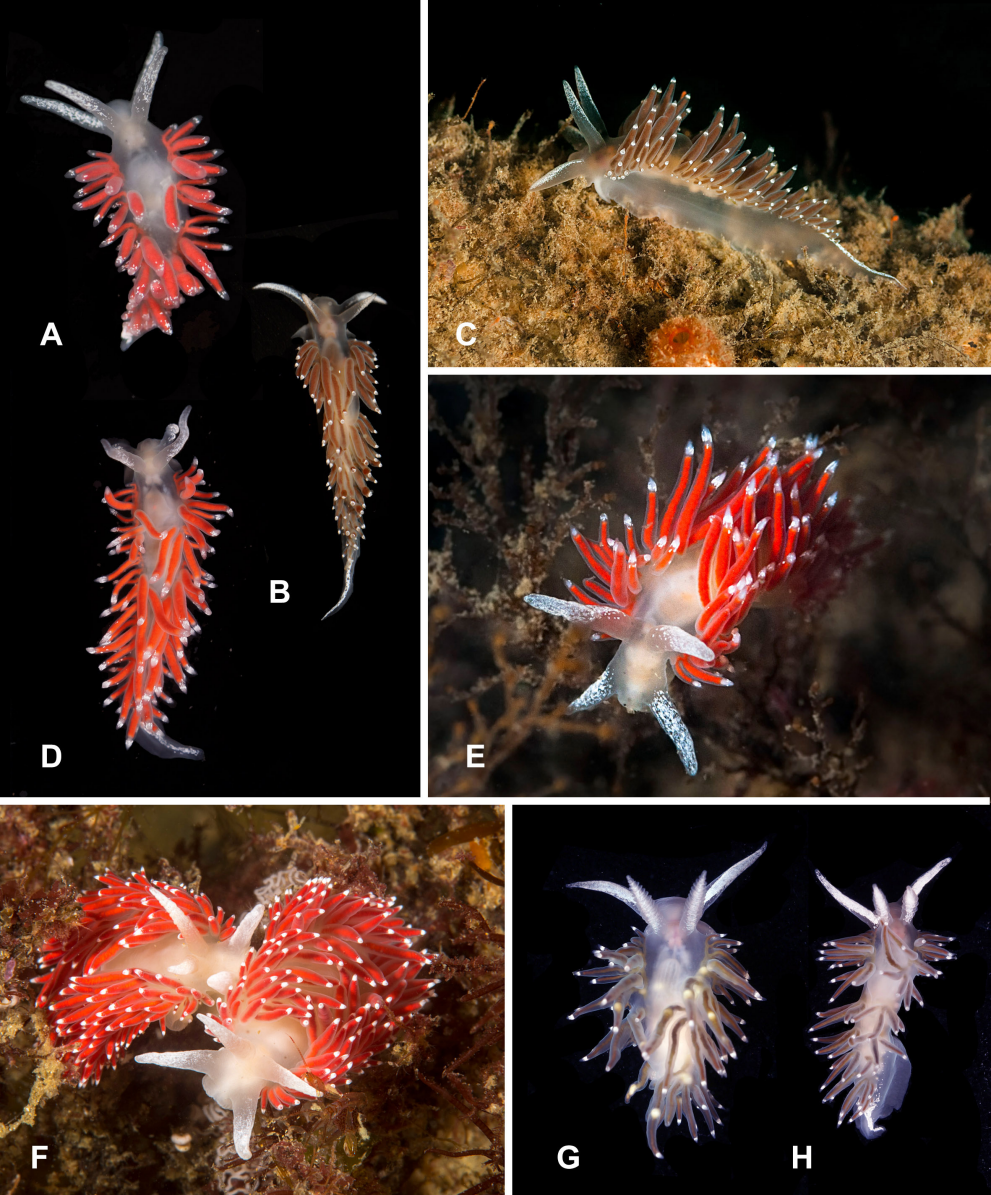
Heterobranchia of surveyed area. (A) *Flabellina* cf. *amabilis*, Klokovo Bay. (B, C) *F. verrucosa*, Klokovo Bay. (D–F) *F. cf. nobilis*, Klokovo Bay. (G, H) *F. trophina*, Dva Brata.

**26. *Flabellina* cf. *amabilis* (Hirano & Kuzirian, 1991)** (Fig. 8A)

*Flabellina amabilis* Hirano & Kuzirian, 1991:48–55, Figs. 1–7.

*“Coryphella” amabilis*—Martynov, 2006; Martynov, 2013.

**Material examined.** 1 specimen, Tretya Langou Bay, 16 m, 4 May 2013, A. Chichvarkhin leg.

**Identification.** Body white semi-translucent. Tiny white dots on oral tentacles, rhinophores, and on cerata below cnidosacs. Cerata with pinky-red appendages of digestive gland.

**Ecology.** Found on sunken rope colonized with *Obelia* hydroids.

**Distribution.** Known from all Russian Pacific seas and Hokkaido shore in Japan (Martynov & Korshunova, 2011).

**27. *Flabellina verrucosa* (Sars, 1829)** (Figs. 8B, 8C)

*Eolidia verrucosa*  Sars, 1829:9–12, pl. 2. Figs. 1–4.

? *Coryphella longicauda* (sic!)—Volodchenko, 1941.

*Coryphella verrucosa*—Martynov, 2013; Martynov & Korshunova, 2011.

*Coryphella pseudoverrucosa* Martynov, Korshunova & Sanamyan, 2015.

**Material examined.** 2 specimens, Tretya Langou Bay, 15 m, 4 May 2013, A. Chichvarkhin leg.

**Identification.** Body white. Cerata brownish-red, never bright red in studied area. White solid stripe on oral tentacle and less solid pigmentation on the rhinophores. Cnidosacs smaller than in similar *C*. cf. *nobilis*. White stripe on tail. Body size to 35 mm.

**Ecology.** In Rudnaya Bay vicinity found on *Obelia longissima* at 12–20 m depth.

**Distribution.** A common species known from all Far Eastern seas of Russia, North America, Arctic and the northwestern Atlantic (Martynov & Korshunova, 2011; Behrens & Hermosillo, 2005).

**28. *Flabellina* cf. *nobilis*  Verrill, 1880** (Figs. 8D–8F)

Verrill, 1880:380.

*Himatina nobilis*—Martynov, 2006; Martynov, 2013.

**Material examined.** 12 specimens, Tretya Langou Bay, 15–18 m, 15 May 2014, A. Chichvarkhin leg.; 1 specimen, Tretya Langou Bay, 15 m, 15 May 2014, A. Chichvarkhin leg.; 1 specimen, Senkina Shapka Pinnacle, 9 m, 14 May 2014, A. Chichvarkhin leg.

**Identification.** Body wide, white. Cerata bright-red. Oral tentacle and the rhinophores are heavily dusted with white pigment. Cnidosacs white, big. White stripe on tail. This is the biggest local *Coryphela* species to 45 mm.

**Ecology.** Occurs on *Obelia* cf. *longissima* hydroids at 10–20 m depth.

**Distribution.** This species is found in Rudnaya Bay, distribution range unknown. *F. nobilis* is known from the northern Atlantic. Similar forms were reported from the Arctic and Pacific seas of Russia (Martynov & Korshunova, 2011), although they may represent several sister species.

**29. *Flabellina trophina* (Bergh, 1890)** (Figs. 8G and 8H)

*Himatella fusca* O’Donoghue, 1921.

*Himatella trophina* Bergh, 1890: 1–75.

*Aeolis camtchatica* Volodchenko, 1941.

*Himatina trophina*—Martynov, 2013; Martynov & Korshunova, 2011.

**Material examined.** 4 specimens, Dva Brata rocks, 5 m, 6 Jun 2013, A. Chichvarkhin leg.; 2 specimens, Dva Brata rocks, 5 m, 16 May 2014, A. Chichvarkhin leg.

**Identification.** Body wide, white semi-translucent. Cerata in continuous rows, brownish, never bright red. White solid stripes on oral tentacle and the rhinophores. Cnidosacs small, white. White stripe on tail. Body size to 25 mm.

**Ecology.** Occurs on rocky walls at 3–6 m depth. Feeds on hydroids.

**Distribution.** The north Pacific seas (Martynov & Korshunova, 2011).

**Remarks.** Martynov (2006) synonymized *Cratena rubra* (Volodchenko, 1941) and *C. trophina*. However, monoserial radula described and drawn by Volodchenko is not specific for Flabelinnidae but characteristic for Tergipedidae. Type specimens of *C. rubra* were collected from soft bottom at 20 m depth—this is unlikely habitat for *C. trophina*, which occurs at shallow depths of 3–6 m on wave exposed rocks. While *Cuthona nana*, which settles on hermit crab shells can easily occur there, moreover, this is the only red colored Tergipedid species known from the Sea of Japan that reach described body length of 25 mm.

**Figure 9:**
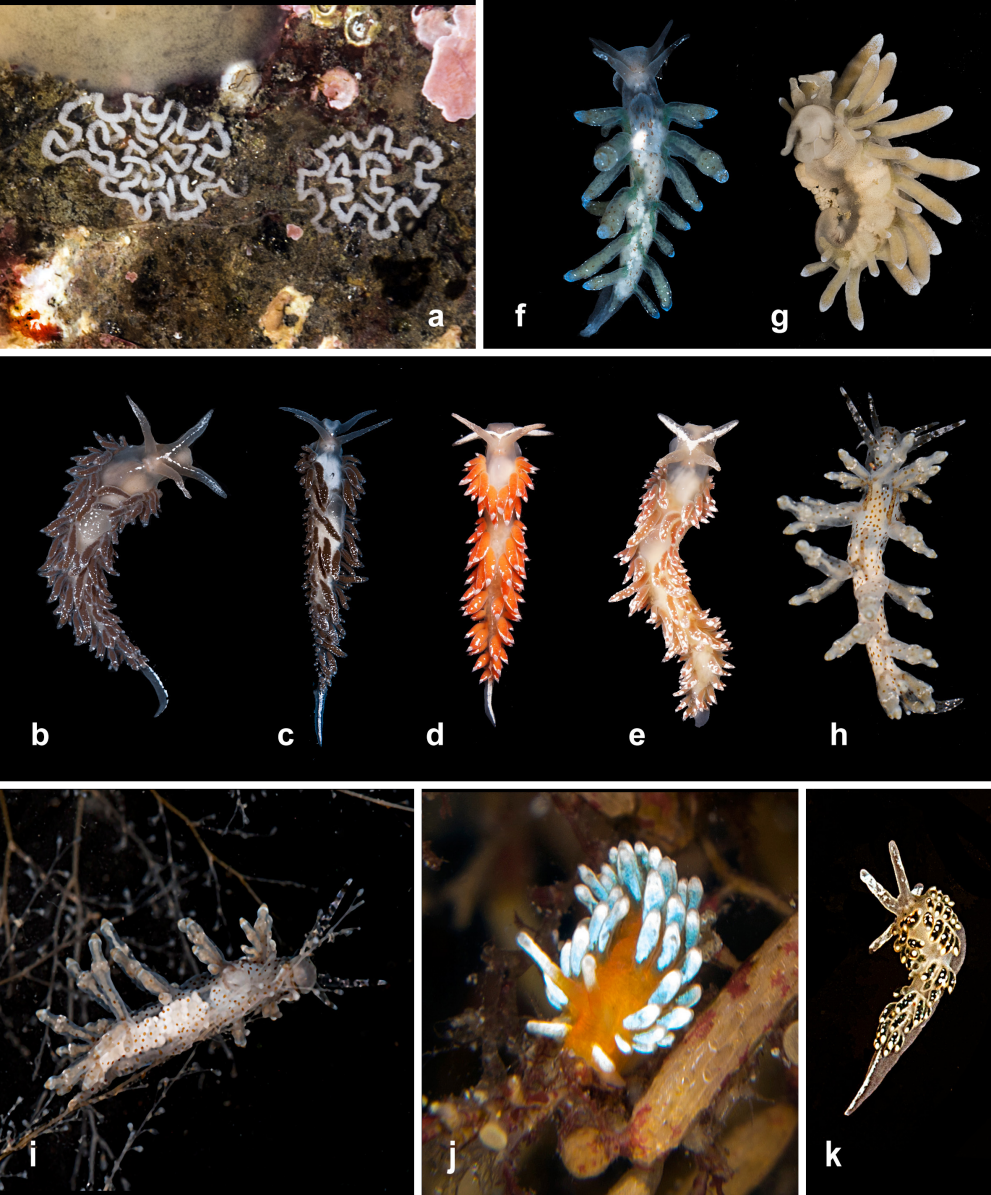
Heterobranchia of surveyed area. (A–E) *Flabellina athadona*, Dva Brata, Klokovo Bay; (F, G) *Eubranchius rupium*, Tretya Langou Bay; (H, I) *E. misakiensis*, Senkina Shapka. (J) *Trinchesia ornata*, Senkina Shapka. (K) *Trinchesia viridis*, Dva Brata.

**30. *Flabellina athadona* (Bergh, 1875**) (Figs. 9A–9E)

*Coryphella athadona*  Bergh, 1875:635-638, pl. 13, Figs. 1–13.

non *Coryphella athadona*—Volodchenko, 1941.

non *Coryphella athadona* (sic!)—Volodchenko, 1955.

*Coryphella athadona*—Martynov & Korshunova, 2011.

*“Coryphella” athadona*—Martynov, 2006; Martynov, 2013.

**Material examined.** 4 specimens, north of Brynner Cape, Rudnaya Bay, 8 m, 4–6 May 2013, A. Chichvarkhin leg.; 2 specimens, Tretya Langou Bay, 16 m, 4 May 2013, A. Chichvarkhin leg.; 2 specimens, Dva Brata rocks, 6–8 m, 14 May 2014, A. Chichvarkhin leg.; 1 specimen, Rudnaya Bay, Brynner Cape, 7 m, 14 May 2015, A. Chichvarkhin leg.; 2 specimens, Vladimir Bay 20 May 2014, K. Dudka leg.; egg masses, Olga Bay, 5 m, 1 Jun 2016, A. Chichvarkhin leg.

**Identification.** Body yellowish-white. Cerata may be colored in various tans on yellow, red and brown. Can be clearly identified with white triangle or X-shaped mark on head and oral tentacles. White stripe on tail. Body size to 20 mm.

**Ecology.** Occurs various substrates at 0–15 m depth, most common on Obelia longissima. Feeds on wide range of hydroids.

**Distribution.** A common species known from all Far Eastern seas of Russia (Martynov & Korshunova, 2011).

**Table:** 

**Superfamily Fionoidea Gray, 1857**
**Family Eubranchidae Odhner, 1934**
**Genus *Eubranchus* Forbes, 1838**
**Type species** *Eubranchus tricolor* Forbes, 1838, by original designation.

**31. *Eubranchus rupium*  Møller, 1842** (Figs. 9F and 9G)

*Tergipes rupium*  Møller, 1842: 78.

*Eubranchus exiguus* –Roginskaya, 1962; Roginskaya, 1987, non Alder & Hancock, 1848.

*Nudibranchus rupium*—Martynov, 1998a; Martynov, 1998b; Martynov, 2006; Martynov & Korshunova, 2011; Yavnov, 2012; Martynov, 2013.

**Material examined.** 2 specimens, Dva Brata Rocks, 4 m, 10 Oct 2015, A. Chichvarkhin leg.

**Identification** Body grey to olive with dark spots and white tiny dots in some specimens. Digestive gland is visible as brown-green reticulate network. The rhinophores translucent, often with white dots and brown ring in the middle point. Oral tentacles two times shorter than the rhinophores. Anterior part of the foot with no appendages. Body size to 13 mm.

**Ecology.** Feeds on *Obelia longissima* and probably other hydroids at 0–20 m depth.

**Distribution.** Widely distributed if Far Eastern seas, Atlantic, and Arctic (Martynov & Korshunova, 2011).

**32. *Eubranchus misakiensis*  Baba, 1960** (Figs. 9H and 9I)

*Aenigmastyletus alexeii*—Martynov, 1998a; Martynov & Korshunova, 2011; Chernyshev, 2014.

**Material examined.** 2 specimens, Vtoraya Langou Bay, 15 m, 16 May 2015, A. Chichvarkhin leg.

**Identification.** Body slim, semi-translucent with clearly separated brownish spots. Digestive gland visible as a brown-green reticulate network. The rhinophores translucent, often with white 2.5-fold longer than oral tentacles. Cerata are swollen in middle part with appropriate local extension of digestive gland. Fore part of the foot with no appendages. Body size to 18 mm.

**Ecology.** Occurs on *Obelia longissima* hydroids at 0–20 m depth.

**Distribution.** Likely, widely distributed in the Sea of Japan.

**Table:** 

**Family Tergipedidae Bergh, 1889**
**Genus** ***Trinchesia*** **Ihering, 1979**
**Type species** *Doris caerulea* Montagu, 1804, by original designation.

**33. *Trinchesia ornata* (Baba, 1937)** (Fig. 9J)

*Cuthona (Hervia) ornata*  Baba, 1937a:331–333, pl. 2, Fig. 4, text-Fig. 17.

**Material examined.** 1 specimen, Senkina Shapka pinnacle, 16 m, 15 May 2014, A. Chichvarkhin leg.; 3 specimens, Senkina Shapka pinnacle, 17 m, 12 May 2015, A. Chichvarkhin leg.; 1 specimen, Senkina Shapka pinnacle, 17 m, 10 Oct 2015, A. Chichvarkhin leg.

**Identification.** Body yellow to orange. Cerata, oral tentacles and proximal parts of the rhinophores white with blue pigmentation in basal part. Body size to 15 mm.

**Ecology.** Occurs on various substrates at 2–20 m depth. Abundant on *Microporina articulata* bryozoan colonies.

**Distribution.** Widely distributed species in the Sea of Japan and Japanese islands (Martynov & Korshunova, 2011).

**34. *Triinchesia viridis* (Forbes, 1840)** (Fig. 9K)

*Montagua viridis*  Forbes, 1840:106–107, pl. 2, Fig. 18)

**Material examined.** 2 specimens, Dva Brata rocks, 4–6 m, 6 Jun 2013, A. Chichvarkhin leg.

**Identification**. Body white. The rhinophores and oral tentacles are translucent, 2/3 proximal part of them is pigmented white. Cerata dusted with white pigment, with brownish-green digestive gland appendages. Cnidosac is distinctive, white under translucent cap. Body size to 15 mm.

**Ecology.** Found on algae covered with the hydroids.

**Distribution.** Widely distributed in the northern Pacific and the northern Atlantic (Martynov & Korshunova, 2011).

**Genus *Cuthona* Alder & Hancock, 1855**

**Type species** *Eolis nana* Alder & Hancock, 1842, by monotypy.

**Figure 10:**
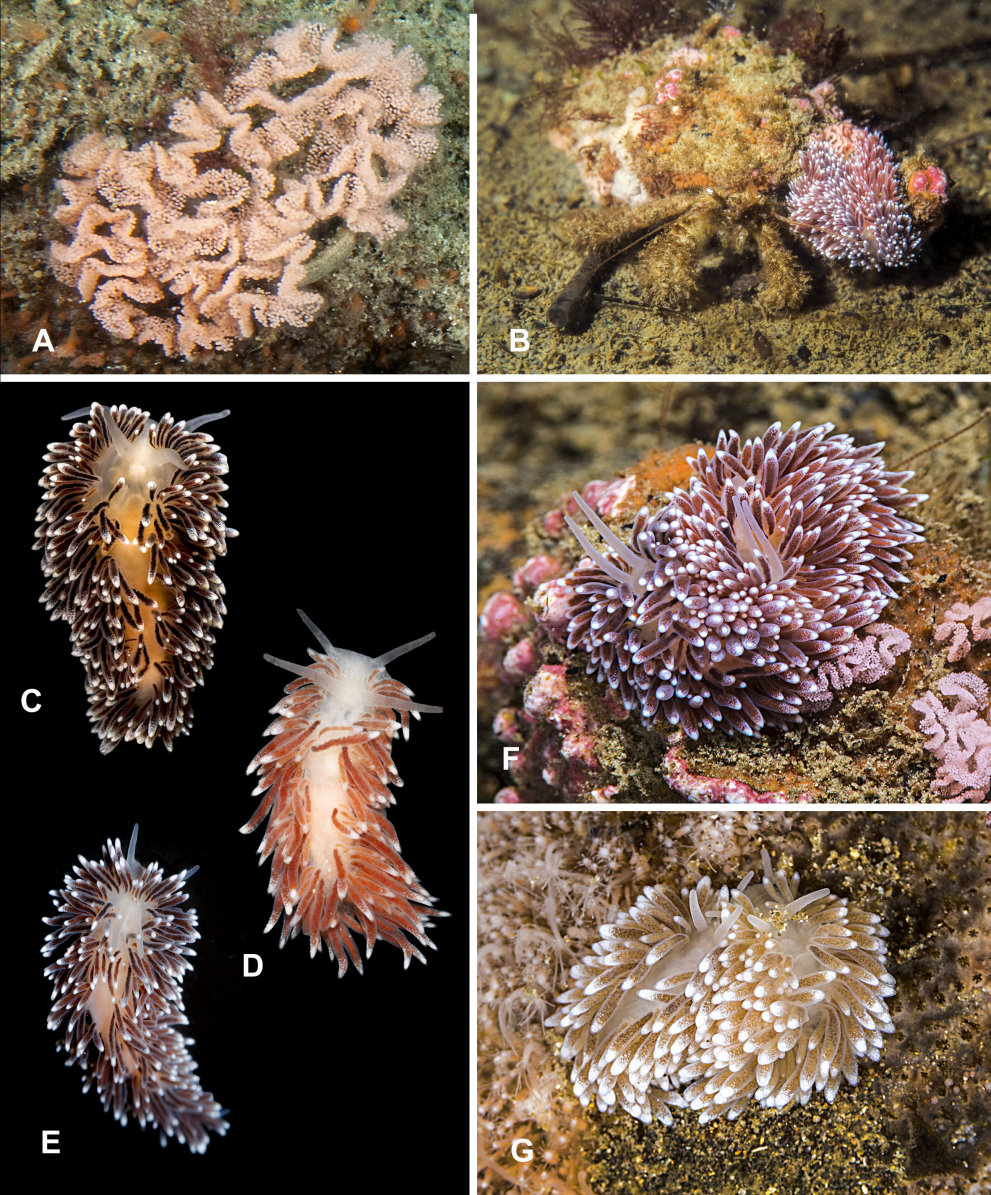
Heterobranchia of surveyed area. (A) *Cuthona nana* egg mass, Brynner Cape. (B, D–G) *C. nana* color forms, Brynner Cape. (C) *C. nana*, Kievka Bay.

**35. *Cuthona nana* (Alder & Hancock, 1842)** (Figs.10A–10G)

*Eolis nana* Alder et Hancock, 1842:31–36.

*Cratena rubra* Volodchenko, 1941.

*Precuthona divae* Marcus, 1961.

*Cuthona sp.*—Nakano, 2004.

*Cuthona hermitophilla* Martynov, Korshunova & Sanamyan, 2015.

non *Cuthona divae*—Nakano, 2004.

**Material examined.** 2 specimens, Rudnaya Bay, Brynner Cape, 6–8 m, 6 May 2013, A. Chichvarkhin leg.; 5 specimens, Rudnaya Bay, Brynner Cape, 6–8 m, 13–16 May 2014, A. Chichvarkhin leg.; 12 specimens, Rudnaya Bay, Brynner Cape, 6–8 m, 15 May 2015, A. Chichvarkhin leg.; 2 specimens, Dva Brata rocks, 6–8 m, 15 May 2014, A. Chichvarkhin leg.; 1 specimen, Kievka Bay, 6–9 m, 29 Jun 2015, A. Chichvarkhin leg.; 2 specimens Rudnaya Bay, Brynner Cape, 6–9 m, 30 May 2016, A. Chichvarkhin leg.; 1 specimen Senkina Shapka Pinnacle, 16 m, 2 Jun 2016, A. Chichvarkhin leg.

**Identification.** Body white semi-translucent. Rhinophores longer than oral tentacles lack pigmentation. Cerata pink with white dots and white cnidosacs. Body length to 30 mm.

**Ecology.** Occurs at the depths of 2–20 m. Feeds on hydroids colonized hermit crabs’ shells. Oviposits on the same shells and hydroid colonies.

**Distribution.** Known from Vladimir Bay, Rudnaya Bay, and Kievka Bay (Chichvarkhin et al., 2016b). Presumably reported from Bering Sea (Martynov & Korshunova, 2011; Martynov, Sanamyan & Korshunova, 2015). Also known from the NE Pacific and Atlantic (Chichvarkhin et al., 2016b).

**Remark.** *Cuthona hermithophila* has been described from Kievka Bay recently. We have thoroughly investigated a population from there and few other populations. All of them are nearly indistinguishable from nominative *C. nana* (Chichvarkhin et al., 2016b).

**Genus *Cuthonella* Bergh, 1884**

**Type species *Cuthonella abyssicola* Bergh, 1884, by monotypy.**

**Figure 11:**
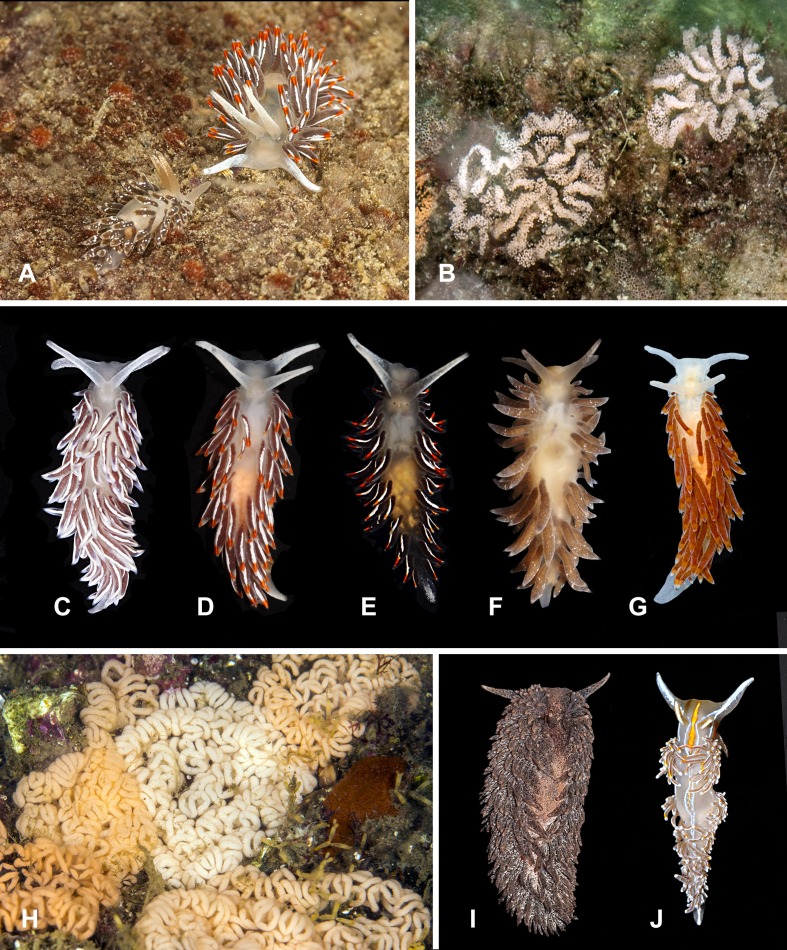
Heterobranchia of surveyed area. (A) *Cuthonella soboli*, Rudnaya Bay. (B) *C. soboli* egg mass, Rudnaya Bay. Color morphs of *C. soboli*: (C, D, F) *Rudnaya Bay*. (E) *Vladimir Bay*. (G) *Vityaz Bay*. (H) *Aeolidia papillosa* egg mass, Senkina Shapka. (I) *A. papillosa*, Senkina Shapka. (J) H*ermissenda crassicornis*, Klokovo Bay.

**36.** *Cuthonella soboli* Martynov, 1992 (Figs. 11A–11G)

Martynov, 1992:18–23, Figs. 1–3.

*Cuthona sp.*—Baba, 1935a; Baba, 1935b; ? Roginskaya, 1964.

*Cuthonella osyoro*—Baba, 1940 (nom. dub.); Martynov, 2006.

*Cuthona cf. punicea*—Nakano, 2004.

**Material examined.** 2 specimens, south of Oprichnik Bay, Viking wreck, 6–8 m, 6 June 2013, A. Chichvarkhin leg.; 5 specimens, Tretya Langou, 16–18 m, 6 June 2013, A. Chichvarkhin leg.; 2 specimens, Brynner Cape, 4 m, 15 May 2014, A. Chichvarkhin leg.; 2 specimens, Dva Brata rocks, 6–8 m, 15 May 2014, A. Chichvarkhin leg.; 2 specimens, Vtoraya Langou, 12–16 m, 16 May 2014, A. Chichvarkhin leg.; 1 specimen, Senkina Shapka pinnacle, 17 m, 15 May 2015, A. Chichvarkhin leg.; 4 specimens, Vladimir Bay 20 May 2014, K. Dudka leg.; 2 specimens, Kievka Bay, 7 m, 29 Jun 2015, A. Chichvarkhin leg.

**Identification.** Maximum body length 20 mm. Body uniformly. Rhinophores and oral tentacles with white pigmentation. Coloration of the cerata varies. Color form from Vladivostok possess brown cerata. Most common form possesses a dark brown colored digestive gland, a white stripe along dorsal side of cerata and orange ring near the tips of cerata. Rare individuals possess no orange pigment or white stripes. A form with orange colored digestive gland, orange pigment with no white stripes is known from Vityaz Bay of the southwestern Peter-the-Great Bay.

**Ecology.** Occurs on various substrates at 0–25 m depth where feeds on wide range of hydrozoans, also fish eggs and presumably *Spirorbis* sp. polychaete.

**Distribution.** Northern part of the Sea of Japan (Martynov & Korshunova, 2011).

**Table:** 

**Superfamily Aeolidioidea Gray, 1827**
**Family Aeolididae Gray, 1827**
**Genus *Aeolidia* Cuvier, 1798**
**Type species** *Limax papillosus* Linnaeus, 1761, by subsequent designation.

**37. *Aeolidia papillosa* (Linnaeus, 1761)** (Figs. 11H and 11I)

*Limax papillosus* Linnaeus, 1761:508.

*Aeolidia papillosa* var. *pacifica* Volodchenko in Ushakov, 1953.

**Material examined.** 2 specimens, Senkina Shapka Pinnacle, 16 m, 13 May 2014, A. Chichvarkhin leg.; 2 specimens, Brynner Cape, 6–8 m, 30 May 2016, A. Chichvarkhin leg.

**Identification.** Body, rhinophores, oral tentacles, and papillae brownish with numerous dots of white pigmentation. Body wide. Size to 70 mm.

**Ecology.** Feeds on *Metridium senile* hexacorals. Occurs on rocks and under stones at 1–20 m depth.

**Distribution.** A member of large amphiboreal cryptic species complex known as *A. papillosa* (Klienberger et al., 2016). The slugs from the Sea of Japan probably constitute a distinct species.

**Table:** 

**Family Facelinidae Bergh, 1889**
**Genus *Hermissenda* Bergh, 1879**
**Type species** *Cavolina crassicornis* Eschscholtz, 1831, by monotypy.

**38. *Hermissenda crassicornis* (Eschscholtz, 1831)** (Fig. 11J)

*Cavolina crassicornis*  Eschscholtz, 1831:15, Fig. 1.

*Aeolis (Flabellina?) opalescens* Cooper, 1863.

**Material examined.** 1 specimen, Vtoraya Langou Bay, 15 m, 7 May 2013, A. Chichvarkhin leg.; 2 specimens, Vtoraya Langou Bay, 16 m, 16 May 2015, A. Chichvarkhin leg.

**Identification.** Body whitish, 30 mm max. Orange line with blue margins along central part of the body. Orange markings on both lateral sides of the head. Long oral tentacles with blue lines.

**Ecology.** A predator that feeds on aeolid nudibranches, mainly on *Flabellina athadona*. Occurs at various depths of 1–15 m depths.

**Distribution.** North Pacific species, occurs from Mexico to Alaska, Sea of Japan, Kurile Islands ((Martynov & Korshunova, 2011); Lindsay & Valdés, 2016).

**Remark.** Recently, Lindsay & Valdés (2016) hypothesized that *H. emurai* (Baba, 1937c) inhabits the western Pacific including Russian waters, while *H. crassicornis* is a NE Pacific species. Although they did not use any materials or data from there for making such a conclusion. The slugs from the Russian waters possess character traits of the ‘northeastern’ *H. crassicornis*: white longitudinal lines on their cerata, which are not arranged in distinct groups, overall coloration brownish, not orange.

## Discussion

The present work updates the knowledge on the scarcely known marine fauna Primorye region; from the 85 species of sea slugs recorded to inhabit Russian waters of the Sea of Japan (Sirenko, 2013; Chichvarkhin, Chichvarkhina & Chernyshev, 2015; Chichvarkhin, Chichvarkhina & Kartavtsev, 2016a; Chichvarkhin et al., 2016d; Martynov, Sanamyan & Korshunova, 2015; Ekimova et al., 2016), the 38 species were recorded in the surveyed region, accounting for about 46% of its sea slug fauna. A large group of species (24) occurring in the area are widely distributed in the northern Pacific Ocean. The eight species are endemic for the Sea of Japan and adjacent part of the Sea of Okhotsk: *Cadlina olgae, Rostanga alisae, Melanochlamys* sp., *Runcinida valentinae, Retusa minima, Cuthonella soboli, Dendronotus dudkai, Eubranchus alexeii*. While seven other species including *Cuthona nana, Eubranchus rupium, Flabellina verrucosa, Dendronotus frondosus, Palio dubia, Clione limacina,* and *Limacina helicina* occur also in northern Atlantic and Arctic waters. Thirteen found species are unknown from Peter the Great Bay but known from the Northern Pacific excluding *Melanochlamys* sp. and *R. valentinae.* Interestingly, several species that are not recorded in the Peter the Great Bay were previously found in the northern Hokkaido, including, e.g., *R. valentinae, J. fuscus,* and *O. muricata*. This fact may detect an introgression pathway of northern species into the Sea of Japan along Kurile Archipelago, Sakhalin, and Hokkaido. Most of studied 38 species can be clearly discriminated using live body shape, size, and coloration, what makes their identification in the field faster and easier. The only problematic group is the genus *Dendronotus,* three species of which (*D. frondosus*, *D. dudkai*, and *D. kamchaticus*) are poorly distinguishable, hence molecular markers or radula examination are preferred for their identification.

## Additional Information and Declarations

### DNA Deposition

The following information was supplied regarding the deposition of DNA sequences: The sequences described here are accessible via GenBank accession numbers KX610756, KX610757, KX610758, KX871888, KX871889, KX938354, KX938355, KX938356, KX938357, KX938358, KX938359, KX938360, KX938361, KX938362.

### Data Availability

The following information was supplied regarding data availability: The research in this article did not generate, collect or analyse any raw data or code.

### New Species Registration

The following information was supplied regarding the registration of a newly described species: Publication LSID: urn:lsid:zoobank.org:pub:02814E3B-C41F-4AA7-80B9- D4DD2ED73FF2; *Cadlina olgae* LSID: urn:lsid:zoobank.org:act:758A5BFF-FDB9-4E19-8D0D-D054358ACE6F.
